# DigestiFlow: from BCL to FASTQ with ease

**DOI:** 10.1093/bioinformatics/btz850

**Published:** 2019-11-14

**Authors:** Manuel Holtgrewe, Clemens Messerschmidt, Mikko Nieminen, Dieter Beule

**Affiliations:** 1 Core Unit Bioinformatics, Berlin Institute of Health, Berlin 10178, Germany; 2 Charité — University Medicine Berlin, Berlin 10117, Germany; 3 Max Delbrück Center for Molecular Medicine in the Helmholtz Association, Berlin 13125, Germany

## Abstract

**Summary:**

Management of raw-sequencing data and its pre-processing (conversion into sequences and demultiplexing) remains a challenging topic for groups running sequencing devices. They face many challenges in such efforts and solutions ranging from manual management of spreadsheets to very complex and customized laboratory information management systems handling much more than just sequencing raw data. In this article, we describe the software package *DigestiFlow* that focuses on the management of Illumina flow cell sample sheets and raw data. It allows for automated extraction of information from flow cell data and management of sample sheets. Furthermore, it allows for the automated and reproducible conversion of Illumina base calls to sequences and the demultiplexing thereof using bcl2fastq and Picard Tools, followed by quality control report generation.

**Availability and implementation:**

The software is available under the MIT license at https://github.com/bihealth/digestiflow-server. The client software components are available via Bioconda.

**Supplementary information:**

Supplementary data are available at *Bioinformatics* online.

## 1 Introduction

Laboratories operating modern-sequencing facilities face a multitude of challenges. These include sample tracking, *raw data pre-processing* (conversion of raw sequencer output into sequences and demultiplexing of pooled experiments which is usually done in the same step), quality control of sequencing results and delivery to the requesting party. Although there is no clear consensus of what comprises a Laboratory Information Management System (LIMS), the term LIMS is often used to describe systems supporting these step. Simple ‘pure peopleware’ implementations consist of spreadsheets on network shares while comprehensive commercial packages such as Illumina BaseSpace Clarity LIMS offer highly adjustable but very expensive solutions. A number of academic and open solutions fall in between, offering a variable number of features and degrees of customizability.

The general lack of agreement of what a LIMS should cover or not cover stems from the fact that sequencing laboratories alone differ greatly. Areas of difference include the types of samples accepted (tissues/blood, DNA/RNA, final libraries/pools or a subset thereof), and the type of data generated (raw base calls (BCLs), sequences, aligned reads or bioinformatics analytical reports). In addition, the surrounding information technology (IT) infrastructure varies greatly as does the degree of integration with such additional IT systems.

In this article we present our approach *DigestiFlow* (DF) that addresses the different needs of organizations by focusing on a small, well-defined subset of tasks: management of Illumina flow cell and sample sheet information and orchestrating the step converting BCLs to sequences and demultiplexing pooled sequencing runs. To the best knowledge of the authors, in this domain DF offers unparalleled functionality. Flow cells can be filled with an arbitrary combination of libraries using any combination of index and molecular barcode reads. DF also supports the barcode being part of the template sequence. DF provides extensive features for sanity checking and comparison of expected indexing reads with those actually seen in the raw BCL data.

This is particularly important in an era where technologies such as single cell and low input sequencing require an ever-growing complexity of barcoding and indexing schemes and the amount of sequencer throughput is growing dramatically. We have encountered flow cells with more than 600 libraries and expect this to grow with increasing sequencer throughput.

A fundamental link to central IT is the integration with existing authentication infrastructure via directory servers, e.g. Microsoft ActiveDirectory (AD). DF supports linking accounts to central AD instances as well as using user accounts that only exist within the system. Beyond this, the system provides its functionality through a REST API (representational state transfer application programmable interface application programing interface) such that other services can be easily integrated. Instead of covering all possible functionality and sample tracking schemes, DF avoids the complexity of a monolithic system and can be integrated as a part of a modular system. However, it can also just be standalone without integration with any other system.

## 2 Materials and methods

**Fig. 1.**
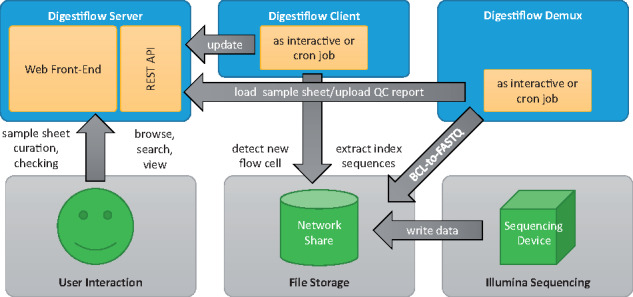
Architectural overview. Sequencing instruments write data to a specified file system storage. A periodically running DF Client detects new flow cells and registers them with the DF Server. Once sequencing is complete and sample sheet information has been approved by the operator, DF Demux performs the conversion to FASTQ files and creates all QC reports. Users can not only browse and view, but also manage and curate flow cells and their sample sheets through DF Server

DF consists of three major components. The architecture of the system is shown in Figure 1. The figure also shows interaction with a minimal set of external systems.

### 2.1 DF server

DF Serverugh being out of scope of this article, we note that DF could be integrated with other software packages as long as they provide an API with additional code. The integration with Parkour LIMS appears particularly appealing as it is based on the same technology as DF (Python/Django) and has few other dependencies itself. Table 1 contains a comparison of the listed tools given some important features.

**Table 1.** Comparison important properties and features in commercial and free software for the management of Illumina flow cells information popular in the sequencing community based

Metric	DigestiFlow	BaseSpace Clarity LIMS	OpenBIS LIMS-ELN	MendeLIMS	MISO	Parkour LIMS
License	MIT	commmercial	free for non-commercial	free for non-commercial	GPL	GPL
Self-hosted	✓	—	✓	✓	✓	✓
LDAP auth	✓	✓	✓	✓	✓	—
(REST) API	✓	✓	✓	—	✓	✓
Sample tracking	minimal	advanced	basic	basic	basic	advanced
Basic pre-proc.	✓	✓	✓	✓	—	✓
Flexible pre-proc.	✓	—	—	—	—	—
Sheet checks	✓	—	—	—	—	—
BCL checks	✓	—	—	—	—	—

pre-proc., preprocessing

### 3.3 Features for improving sequencing results

#### 3.3.1 Sample sheet validation

**Fig. 2.**
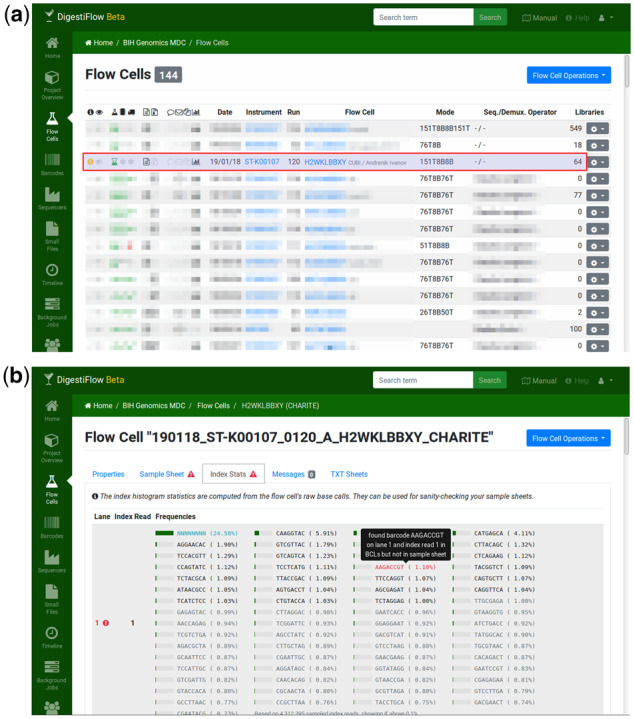
When adding the sample sheet (not shown), the operator made a small mistake. The adapter P37 is given twice for the same lane in the sample sheet while the adapter sequence ‘AAGACCGT’ occurs in the raw BCLs but not in the sample sheet. This information can then be used for debugging sample sheet information. This is highlighted in the sample sheet **(a)** and the display of the adapters read from the raw BCL data **(b)**

Based on practical experience, we greatly appreciate the automated comparison of observed adapter sequence content and sample sheet. Unexpected sequence in either set is an indication for possible errors. DF Server provides fine-grained control to acknowledge and suppress inconsistency warnings (after either fixing errors or accepting errors and then excluding corresponding data). Furthermore, common artifacts such as PhiX sequence are automatically recognized and show up as information rather than warnings or errors. Figure 2 shows an example.

#### 3.3.2 Reproducibility, automation and quality control

The DF Client and Demux components are available from Bioconda as Conda packages and Docker images, thus allowing for future proof installations and creating reproducible workflows. By offering REST APIs and two useful client applications, DF greatly supports sequencing and demultiplexing operators in automating their work. Further automation can be added later as the APIs are open. Automated quality control using FastQC and aggregation using MultiQC also allows users to spot problems earlier (together with the sample sheet adapter checks described earlier). In our experience this allows for the early detection of many common issues. For example, from time to time, it occurs that the same adapter was used for two different libraries in the same lane. This error might be hard to spot on paper or in spreadsheets but applications such as DF Server can easily detect and report such problems similar to the example shown in Figure 2.

## Supplementary Material

btz850_Supplementary_Data
