# Supplementary material for: DigestiFlow: from BCL to FASTQ with ease
Source: Bioinformatics. 2019 Nov 14;36(6):1983–5. doi: 10.1093/bioinformatics/btz850 (PMC7703778; doi:10.1093/bioinformatics/btz850)
Supplement: btz850_Supplementary_Data [file btz850_supplementary_data.pdf]

Digestiflow Server Documentation

Release 1.0.0+0.g3afc1f5.dirty

Manuel Holtgrewe

May 07, 2019

Contents:

1 Installation **3**
- 1.1 Prerequisites . . . 3
- 1.2 Overview . . . 3
- 1.3 Install with Docker Compose . . . 4
- 1.4 Manual Installation . . . 5
- 1.5 Deploy to Heroku . . . 7

2 Tutorial: Overview **9**
- 2.1 Processes Around Sequencing . . . 9
- 2.2 Step 1: Sequencing . . . 9
- 2.3 Step 2: Demultiplexing . . . 10
- 2.4 Step 3: Data Delivery . . . 11

3 Tutorial: Setup Sites **13**

4 Tutorial: Configure Sequencers **15**

5 Tutorial: Configure Barcodes **19**

6 Tutorial: Flow Cells **23**
- 6.1 Manual Flow Cell Creation . . . 23
- 6.2 Automated Flow Cell Creation . . . 24

7 Tutorial: Fill Out Flow Cell **29**
- 7.1 The Sample Spreadsheet . . . 29
- 7.2 Transfer into Digestiflow . . . 29

8 Tutorial: Demultiplex Flow Cell **31**
- 8.1 Installing Digestiflow Demux . . . 31
- 8.2 Performing the Demultiplexing . . . 31

9 Permissions **35**
- 9.1 Member Roles . . . 35
- 9.2 Member List . . . 36
- 9.3 Managing Memberships . . . 36
- 9.4 Sending Invites . . . 36
- 9.5 Django Admin . . . 36

10 Instance-Wide Search **37**

11 Sequencers **39**
11.1 Sequencer List 39
11.2 Sequencer Details 39

12 Barcodes **41**
12.1 Barcode Set List 41
12.2 Barcode Set Details 41
12.3 Updating Barcode Sets 42

13 Flow Cell Overview **43**
13.1 Run Directories 43
13.2 RTA Files 44
13.3 Flow Cell Records 44
13.4 Cycle Information 44

14 Flow Cell Views **45**
14.1 Flow Cell Details 45
14.2 Flow Cell List 47
14.3 Updating Status 47

15 Flow Cell Editor **49**
15.1 Editing Libraries 49
15.2 Library Copy & Paste 50

16 Flow Cell Messages **51**

17 Admin Alerts **53**

18 API Tokens **55**
18.1 Listing API Tokens 55
18.2 Creating API Tokens 55

19 API Access **59**
19.1 First Examples 59
19.2 Authentication 60
19.3 Object Identifiers 60
19.4 API Patterns 60
19.5 Available API Endpoints 61

20 Contributors **63**

21 History / Changelog **65**

DigestiFlow is a suite of programs for managing Illumina flow cells, curating sample sheets, and demultiplexing. **Digestiflow Server** is the database system offering a web-based user UI and REST API.

Note: The Digestiflow Server documentation is currently under construction and in **beta** state. Don't hesitate to open issues in our GitHub bug tracker in case of any question or problem.

CHAPTER 1

Installation

This section describes the installation of Digestiflow, in particular Digestiflow Server. Digestiflow Server provides the database for the flow cells and barcodes as well as the REST API. As a server component, it is the most complex component of Digestiflow Suite to setup. You will be able to install the client components Digestiflow CLI and Digestiflow Demux as standalone (Bio-)conda packages which will be considerably easier.

Overall, Digestiflow Server is a Twelve-Factor App which means that it is fairly easy to deploy (as modern web applications go) but you will have to fulfill a few prerequisites. Further, we will outline the specific installation and configuration steps below. In the Digestiflow Server repository, you will also find a directory `ansible` which contains some Ansible playbooks that you can base your own Ansible playbooks for the installation on. It should be easy convert this into your existing server management infrastructure (Ansible, Puppet, Salt, . . .)

1.1 Prerequisites

You will need to have the following prerequisites fulfilled.

Linux/Unixoid Operating System The following instructions have been tested on Linux. Your mileage might vary for Mac or Windows Linux Subsystem etc.

Linux/Unix Experience Intermediate knowledge about managing a Linux/Unix system will be required.

Python 3, >=3.5 Digestiflow Server is a Django-based web server and requires Python 3.

Python Virtualenv You should be able to run the `virtualenv` command. Consult your operating system's or Python distribution's manual on how to do this.

PostgreSQL, >=9.5 A modern PostgreSQL installation is required for storing the data.

Redis A key/value store required for caching and task queues.

1.2 Overview

In the following, we will describe how to either

1. install via Docker Compose **for testing Digestiflow (recommended)**, or
2. perform a manual installation on a Linux server
   (a) setup a PostgreSQL user for the web app,
   (b) install the web app and setup a virtualenv environment for it,
   (c) configure the web app, and
   (d) initialize the database and create a super user account, or
3. deploy to the Heroku platform (for free but a credit card is required for registration.

Afterwards, you should follow through the tutorial and then explore the rest of the documentation to see the full feature set of Digestiflow.

1.3 Install with Docker Compose

If you have not done so yet, follow the *Docker CE installation instructions <https://docs.docker.com/install/>*_ for installing Docker itself.

1.3.1 Install Docker (Compose)

E.g., on CentOS

```
$ sudo yum remove docker docker-client docker-client-latest docker-common docker-
↪latest docker-latest-logrotate docker-logrotate docker-engine
$ sudo yum install -y yum-utils device-mapper-persistent-data lvm2
$ sudo yum-config-manager --add-repo https://download.docker.com/linux/centos/docker-
↪ce.repo
$ sudo yum install docker-ce docker-ce-cli containerd.io
$ sudo systemctl enable docker
$ sudo systemctl start docker
$ sudo curl -L "https://github.com/docker/compose/releases/download/1.24.0/docker-
↪compose-$(uname -s)-$(uname -m)" -o /usr/local/bin/docker-compose
$ sudo chmod +x /usr/local/bin/docker-compose
```

or on Ubuntu:

```
$ sudo apt-get remove docker docker-engine docker.io containerd runc
$ sudo apt-get update
$ sudo apt-get install apt-transport-https ca-certificates curl gnupg-agent software-
↪properties-common
$ curl -fsSL https://download.docker.com/linux/ubuntu/gpg | sudo apt-key add -
$ sudo add-apt-repository "deb [arch=amd64] https://download.docker.com/linux/ubuntu
↪$(lsb_release -cs) stable"
$ sudo apt-get update
$ sudo apt-get install docker-ce docker-ce-cli containerd.io
$ sudo curl -L "https://github.com/docker/compose/releases/download/1.24.0/docker-
↪compose-$(uname -s)-$(uname -m)" -o /usr/local/bin/docker-compose
$ sudo chmod +x /usr/local/bin/docker-compose
```

1.3.2 Digestiflow Server with Docker Compose

First, checkout the source code

```
$ git clone https://github.com/bihealth/digestiflow-server.git
```

Next, simply call *sudo docker-compose up* in the *docker* sub folder of your setup

```
$ cd digestiflow-server/docker
$ sudo docker-compose up
Creating network "docker_db_network" with driver "bridge"
Creating network "docker_nginx_network" with driver "bridge"
Creating volume "docker_db_volume" with default driver
Pulling db (postgres:9.6)...
9.6: Pulling from library/postgres
27833a3ba0a5: Pull complete
[...]
web_1    | [2019-04-10 21:04:58 +0000] [1] [INFO] Starting gunicorn 19.9.0
web_1    | [2019-04-10 21:04:58 +0000] [1] [INFO] Listening at: http://0.0.0.0:8000␣
↪(1)
web_1    | [2019-04-10 21:04:58 +0000] [1] [INFO] Using worker: sync
web_1    | [2019-04-10 21:04:58 +0000] [79] [INFO] Booting worker with pid: 79
```

You can now log into Digestiflow Server through the following URL (ignore the security warning for the self-signed SSL certificate):

- https://localhost:8443/

You can login with user name *root* and password *root*.

1.4 Manual Installation

The following assumes a CentOS 7.4 system but you should be able to adjust it to any modern Linux distribution.

First, install the required packages.

```
### install EPEL repository
$ yum install -y epel-release
### install IUS repository and packages
$ yum install -y https://centos7.iuscommunity.org/ius-release.rpm
$ yum install -y python36u python36u-pip python36u-devel python36-upsycopg2
### install Postgres repository and packages
$ yum install -y https://download.postgresql.org/pub/repos/yum/9.6/redhat/rhel-7-x86_
↪64/pgdg-redhat96-9.6-3.noarch.rpm
$ yum install -y postgresql96-server postgresql96-devel postgresql96-contrib
```

1.4.1 PostgreSQL Setup

Creating a user and database through the `createuser` and `createdb` commands is easiest. You have to do this as the `postgres` user. We're using `digestiflow_server` both for the user name and password. You should pick a better password!

```
$ sudo -u postgres createuser -E digestiflow_server
Enter password for new role: digestiflow_server
Enter it again: digestiflow_server
$ createdb -l UTF-8 -O digestiflow_server
```

You have now setup a database `digestiflow_server` owned by the user `digestiflow_server`.

1.4.2 Install Web App

Installation of the web app is very simple, you just clone it source code via git. The following will get the latest stable version from branch `master`:

```
# git clone https://github.com/bihealth/digestiflow-server.git
```

Next, create a virtual environment with the dependencies for running it in production mode.

```
# virtualenv -p python3 digestiflow-server-venv
# source digestiflow-server-venv/bin/activate
(digestiflow-server-venv) # cd digestiflow-server-venv
(digestiflow-server-venv) # pip install -r requirements/production.txt
[...]
```

Once this is complete, you are ready to configure the web app.

1.4.3 Configure Web App

All of Digestiflow Server can be configured as environment variables as is common for a Twelve-Factor App. This has the advantage that you do not have to touch Digestiflow Server's source code and all configuration can be done outside it (e.g., in a `systemd` environment file as shown in the Ansible files shipping with the source code).

The following shows a set of the available environment variables, the required ones are marked with `#**`. Put the following into a file `.env` in your `digestiflow-server` checkout and adjust it to your liking and requirements.

```
# Disable debugging (is default)
DJANGO_DEBUG=0

#** PostgreSQL configure user:password@host/database_name for PostgreSQL connection
DATABASE_URL="postgres://digestiflow_server:digestiflow_server@127.0.0.1/digestiflow_
↪server"

#** Use production settings
DJANGO_SETTINGS_MODULE=config.settings.production
#** Configure secret key for session etc.
DJANGO_SECRET_KEY=CHANGE_ME!!!

# Configuration for sending out emails
EMAIL_SENDER=CHANGE_ME@example.com
EMAIL_URL=smtp://CHANGE_ME.example.com
EMAIL_SUBJECT_PREFIX="[Your SODAR Django Site]"

# You can enable LDAP authentication for up to two different sites.  See
# django-auth-ldap documentation for more details.
ENABLE_LDAP=0
AUTH_LDAP_SERVER_URI=
AUTH_LDAP_BIND_PASSWORD=
AUTH_LDAP_BIND_DN=
AUTH_LDAP_USER_SEARCH_BASE=
AUTH_LDAP_USERNAME_DOMAIN=
AUTH_LDAP_DOMAIN_PRINTABLE=

ENABLE_LDAP_SECONDARY=0
AUTH_LDAP2_SERVER_URI=
AUTH_LDAP2_BIND_PASSWORD=
```

(continues on next page)

(continued from previous page)

```
AUTH_LDAP2_BIND_DN=
AUTH_LDAP2_USER_SEARCH_BASE=
AUTH_LDAP2_USERNAME_DOMAIN=
AUTH_LDAP2_DOMAIN_PRINTABLE=

# Configuration for SODAR-core projectroles app
PROJECTROLES_SEND_EMAIL=1
PROJECTROLES_SITE_MODE=TARGET
PROJECTROLES_TARGET_CREATE=1
#** Name of the super user, adjust if you change the superuser name below.
PROJECTROLES_ADMIN_OWNER=admin

#** Configure URL to Redis, this is for a default Redis installation
CELERY_BROKER_URL=redis://localhost:6379/0
```

Once complete, you can use the following to create a admin/super user. Make sure that you have your virtualenv activated.

```
# python manage.py createsuperuser
[follow on-screen instruction]
```

Once you have completed this step, you can use the following command for starting up the server. Do this and log in as the super use you just created.

```
# python manage.py migrate
# python manage.py collectstatic
# python manage.py runserver
[now direct your browser to the displayed URL and login]
```

1.5 Deploy to Heroku

The easiest way to try out Digestiflow Server is to click Deploy to Heroku (or the button on the Github project README) and follow the step-by-step process.

1. Create an account and sign in if necessary.
2. Set the application name, e.g., to `my-digestiflow-demo`.
3. **Click Deploy app and. . . wait a bit.**
    - It seems that, sadly, you will have to enter credit card for account verification. Note that you can try out Digestiflow Server with free/hobby plan only. Also, this is a bit unintuitive, deployment will fail. You have to enter your credit card information and then continue. . .
4. After deployment has succeeded, go to `Manage App`, then `Settings` in the Heroku Dashboard. There, click `Reveal Config Vars` and copy the value after `DIGESTIFLOW_INITIAL_ROOT_PASSWORD` into your clipboard.
5. Go to https://my-digestiflow-demo.herokuapp.com/login/ and login as *root* with the root password copied above.
6. Finally click the little user icon on the top left and then *Admin*. Here you can change the root user's password, create new users etc.

From here, you can read the built-in manual at https://my-digestiflow-demo.herokuapp.com/manual/ (or clicking "Manual" in the right of the top navigation bar).

CHAPTER 2

Tutorial: Overview

This section gives an overview of the "mental model" behind the process around sequencing and demultiplexing from the point of view of Digestiflow. It will describe the boundaries of the Digestiflow system and give examples what it does not attempt to support you with. Further, it will introduce the different components of Digestiflow and how they support you in the sequencing and demultiplexing processes.

2.1 Processes Around Sequencing

The Digestiflow process model starts with the *sequencing* of *libraries* on certain *lanes on a flow cells*. Digestiflow does **not**, e.g., consider your experimental design, help you in sample tracking, or with the storage or organization of your raw BCL or your produced FASTQ sequencing files. It does also not help you with processing these files.

The authors consider such processe **external** to Digestiflow. However, Digestiflow offers a REST API that can be used to connect external software components to the Digestiflow system. For example, you could use the API to automatically fill the sample sheets based on the output of a barcode reader, or start bioinformatics processing such as read mapping. It will be your task to integrate Digestiflow into your external processes. Setting such clear boundaries allows Digestiflow to stay in a sweet spot of relative ease of use but also flexibility and allowing for integration with external services.

Overall, Digestiflow considers three steps:

1. Sequencing
2. Demultiplexing (or base call processing, e.g., to sequences or to base call archives)
3. Data Delivery

2.2 Step 1: Sequencing

Generally, we find that *state machines* are a useful way to describe finite processes. The Digestiflow Server component keeps track of your flow cell sequencing runs, the libraries thereon, and their states. The states for sequencing are:

initial The system knows about the sequncing of a flow cell but the sequencing has not started yet.

running The sequencing device has started with the sequencing process.

complete The sequencing device finished and indicates that it detected no errors.

failed The sequencing device finished and indicates that it detected an error.

confirmed complete A human operator has inspected the sequencing output (e.g., the Illumina RTA and SAV results) for quality control (QC). They then deemed the results of passing QC.

confirmed complete with warnings A human operator has inspected the output and found some issues. The result is not considered a full QC failure but the results have to be treated with caution.

confirmed failure After inspection of the sequencing results, the operator decides that the results are generally unfit for future processing. Any further consideration, if any, of the data has to be done with extreme caution.

The *start states* are initial and running. The initial state is triggered by a user manually registering the flow cell before the system has gained knowledge over a sequencing run. This is done by entering it in the Digestiflow Server UI.

The running state is usually triggered by the Digestiflow CLI component. This software can be setup as a watcher process and periodically scans the storage volume that the sequencing machine writes to. As soon as it sees a new flow cell directory, it will register it with the Digestiflow Server API with the information that it can extract automatically. In the case that the flow cell already exists, it will still update the flow cell information from the flow cell directory through the Digestiflow Server API, e.g., keeping track of the current cycle, or updating missing or incorrectly filled fields.

Digestiflow CLI then also keeps track of the further sequencing state as indicated by the status files in the flow cell directory. By doing this, it is able to detect success (a marker file is created) and certain failures. However, for certain critical technical failures such as issues with the storage volume or power failure in the sequencing lab cannot be easily detected. Further, Digestiflow CLI will analyze the index adapters once their sequencing is complete and register statistics with Digestiflow Server UI. This way, mismatches of the adapter base calls with the sample sheet can be detected already during sequencing.

Finally, Digestiflow CLI will update the state to complete or failed from which a human operator has to manually switch the sequencing to one of the final "confirmed" states.

2.3 Step 2: Demultiplexing

A filled sample sheet is the precondition for the demultiplexing of data. This can be done after the sample sheet is known to the system, either by being added manually, or by being registered through Digestiflow CLI. Once the sheet has been filled, the demultiplexing can start using Digestiflow Demux. This component is also setup as a second watcher process to consider the sequencer run directories.

The demultiplexing states are as follows:

initial This is the default initial state.

ready After filling the sample sheet, the operator manually updates the state to "ready". This marks the flow cell to be ready for demultiplexing once the sequencing state is "complete" or one of the confirmed states.

running The demultiplexing process has been marked as running after being started.

complete The demultiplexing process has finished successfully and Digestiflow Demux has marked the state as "complete".

failed The demultiplexing process has failed for some reason and Digestiflow Demux has marked the state as "failed".

confirmed complete The demultiplexing operator has performed quality control of the demultiplexing results and considers them to pass the quality control.

confirmed failure The demultiplexing operator is unable to perform the demultiplexing correctly and marks it as failed.

Note that there is no “confirmed warning” state as this step assumes that discordances between the sample sheet and the libraries are resolved in the sample sheet. By removing missing adapters from the sample sheet (and thus adjusting it to the experimental reality), and/or acknowledging any further mismatches, the sample sheet is put into consistency with the base calls.

Also note that Digestiflow offers to create tarball archives of the lanes instead of or in addition to the demultiplexing. This is necessary as some sequencing facility customers/users prefer to start their pipelines from the BCL files. Creating tarballs of individual lanes simplifies the extraction of the necessary per-flowcell and the desired per-lane information into one archive file.

2.4 Step 3: Data Delivery

Data delivery is considered a manual process. The delivery operator performs the communication with the data recipient, sends the data in an appropriate way (e.g., a shared network volume), and finally confirms that the recipient received the data and the delivery is over.

initial Delivery does not have started.

in progress Files are being transferred or the operator is waiting for the recipient to confirm receiving the data.

complete The files have been delivered successfully.

skipped Delivery has been skipped as it is unnecessary, e.g., for test data internal to the sequencing facility.

Read on in the tutorial to learn about how Digestiflow supports you in these processes.

CHAPTER 3

Tutorial: Setup Sites

Digestiflow can support more than one sequencing lab per installation by managing a set of **sites**. After logging into Digestiflow with the super user that you created in the installation step, you first have to setup a first site by clicking on “Create Site” in the left green action bar. If your window is not wide enough, that link might be hiding in a dark-gray symbol with three stripes. It might be a good idea to zoom out a bit in your browser window so you see the green action bar on the left-hand side.

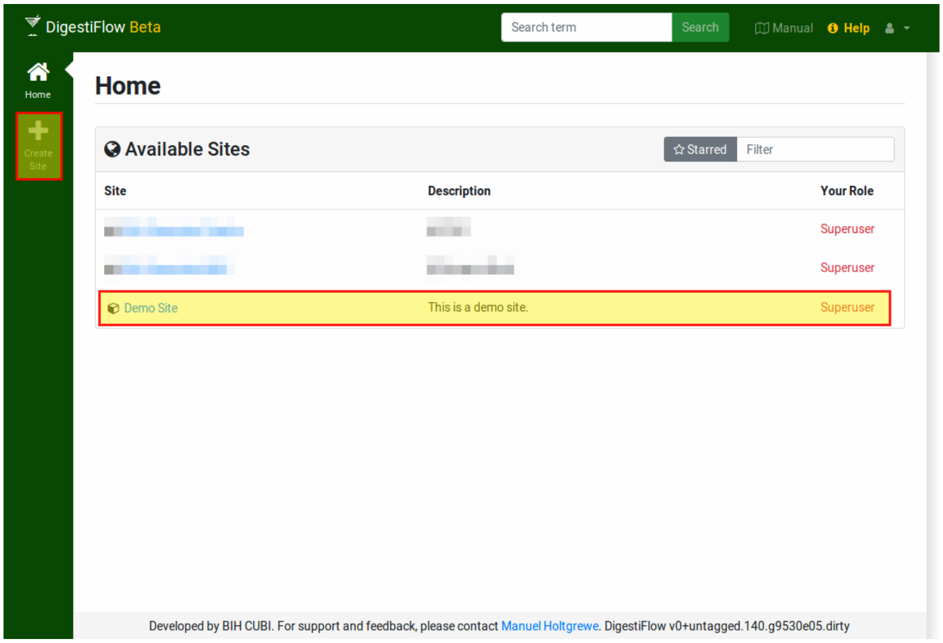

Fig. 1: The site list as displayed when logged in as a super user. The “Create Site” icon on the left-hand bar allows the creation of new sites.

Fill out the “Create Site” form’s Title and Description field, you can leave the remaining ones at their default settings. After creating the site, you will navigate to it and you will see its overview page. The main part on the right will give the title, a short description/README, the latest five flow cells, the latest five added barcode sets, sequencing

machines. Further, as an admin of the site, you will see the latest five small files (created from message attachments), and the latest fives timeline entries.

Read on to learn about setting up sequencers and barcode sets.

CHAPTER 4

Tutorial: Configure Sequencers

First, you have to register the sequencers of your lab with Digestiflow. For this, click the "Sequencers" section in the left-hand green icon bar. You will are then redirect to a site listing all of your sequencers.

Click the blue button "Sequencer Operations" button on the top right and then "Create". It is important to configure the machine model and the "Dual index workflow" appropriately such that demultiplexing works correctly with dual indexing. Fill the form and click the "Create" button on the bottom right to create it.

If you want to follow the tutorial and add a flowcell with simulated data, you can now add a sequencer of model Hiseq 2000 called "CSSIM".

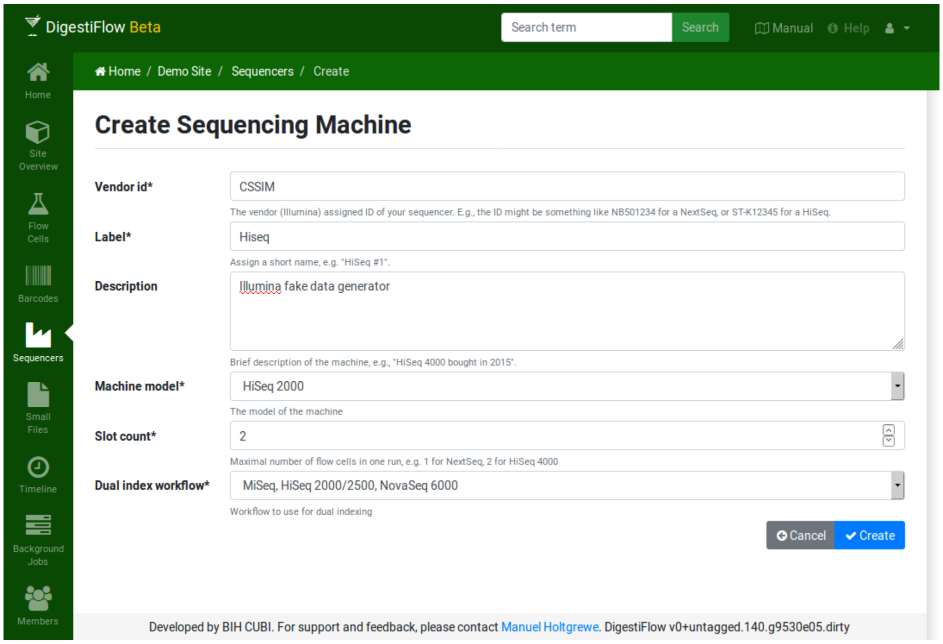

Fig. 1: The form for creating a sequencing machine.

After creating the sequencer you are redirected to the detail view of the sequencer.

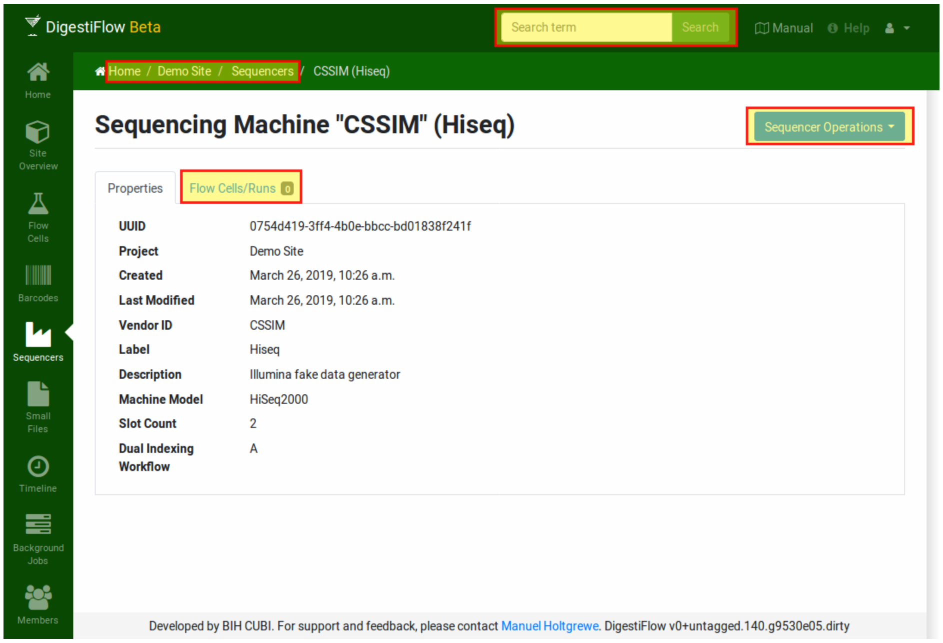

Fig. 2: The detailed view of the sequencer.

The "Properties" tag shows the details of the sequencer. For example, the sequencer reecord that we just created has two "slots" and allows to sequence using two flow cells at the same time. The "Flow Cells/Runs" tab displays all runs - at the moment no runs, a count of 0. The blue button on the top right gives access to updating or deleting the flow cell record, or exporting it to JSON.

Note that using the "breadcrumb" bar on the top allows for navigating back to the sequencer list or even the site overview. Also note that you could now already use the search function in the top bar and, e.g., entering "cssim" (or even "css") would find your sequencer. Next, use the breadcrumb bar to navigate back to the list of sequencers.

In the list of sequencers, you can click the sequencer ID to see the sequencer details. The table of sequencers has a small gray button with a cog icon. Click it to see the actions for the sequencer (e.g., delete or update).

Read on how to add a barcode set and create a flow cell.

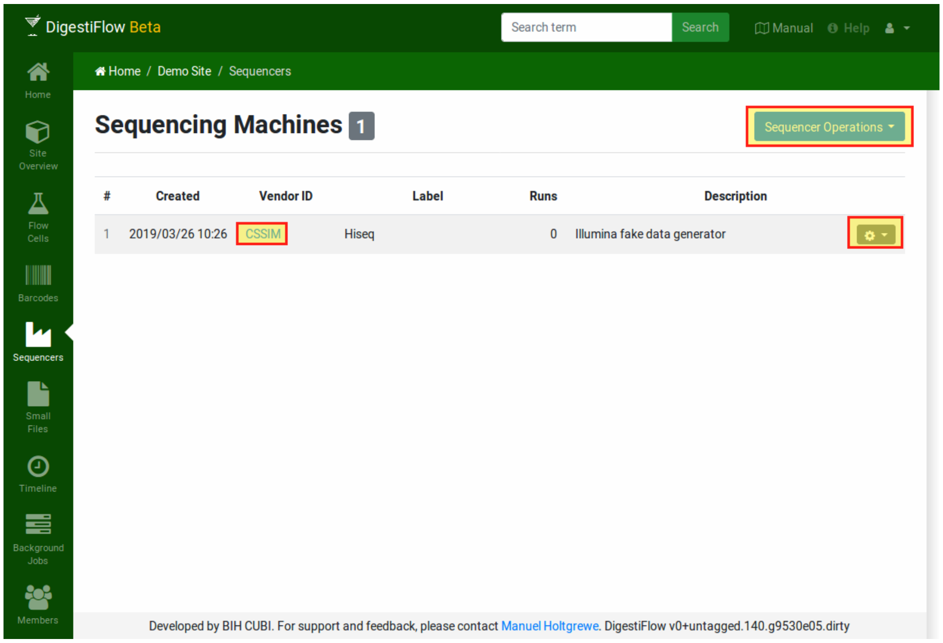

Fig. 3: The sequencer list view.

CHAPTER 5

Tutorial: Configure Barcodes

Clicking the "Barcodes" entry in the green menu on the left-hand brings you to the barcode sets overview of your site. This allows for managing barcode (index adapter sequence) sets in your site that you can reuse. You can then refer to these barcodes by their name (or number) and thus simplify the error-prone copy-and-paste step of copying adapter sequences around.

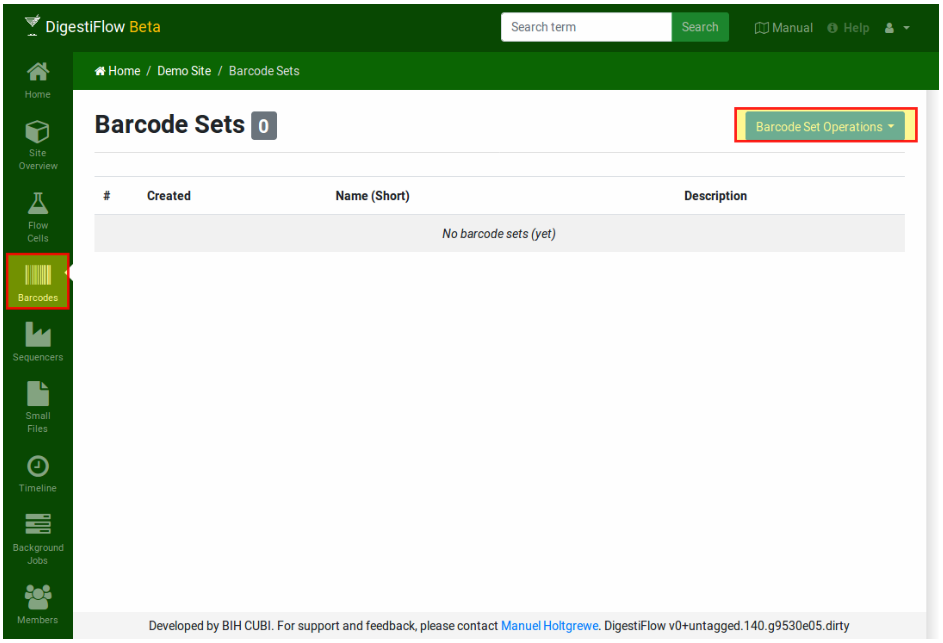

Fig. 1: An empty list of barcode sets.

Managing barcodes is similar to managing sequencers. First, click the blue "Barcodes Operation" on the top right and then "Create".

Fill in the basic properties of the barcode set in the "Properties" page.

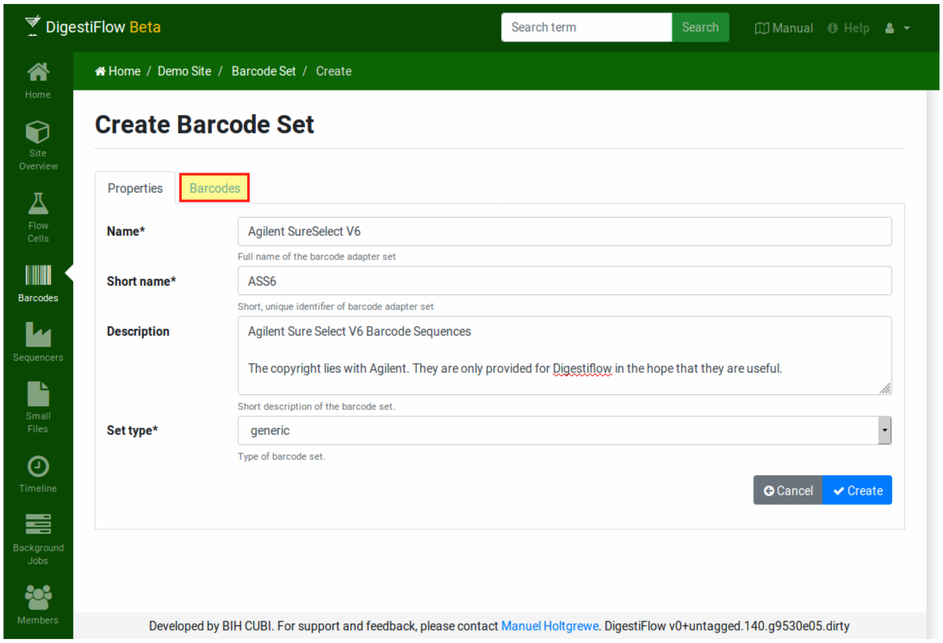

Fig. 2: Editor for the barcode set properties.

Then, select the "Barcodes" tab. In case of display errors of the table, just click in the first cell of the leftmost column. You can now enter (or better: copy and paste) your barcodes from a spreadsheet or your kit vendor's manual. You can find an example for the Agilent SureSelect V6 adapters in the file `Barcodes_Agilent_SureSelect_V6.xlsx` in here.

Simply copy and paste the data from the spreadsheet file into the barcodes table. Note that a context menu is available, e.g., for quickly reverse-complementing bases.

The columns of the are as follows:

name The primary name of the adapter, e.g., `A001`.

aliases An optional, comma-separated list of adapters names, e.g., `1,01,001`.

sequence The adapter sequence. Note that you always enter the **forward** adapter sequence in Digestiflow. In the case of dual indexing, Digestiflow Demux will automatically reverse-complement the adapter sequence if necessary for the dual indexing workflow of your sequncing device.

status This field is updated by the barcode editor and indicates whether the current row is added or changed.

After completely filling out the barcode set table, continue by clicking the blue "Create" on the top right of the form. The detail screen of a barcode set offers a blue "Barcode Set Operations" button which gives access to creating new barcode sets, updating or deleting the current one, or creating a JSON data export.

Again, the breadcrumb on the top allows you to quickly navigate to the barcode set list or the project overview. Both will show the barcode set that you just created.

Before proceeding to the creation of flow cells, add a new barcode set for test data. You can find the barcode set properties and sequences in the file `Barcodes_Test.xlsx` here.

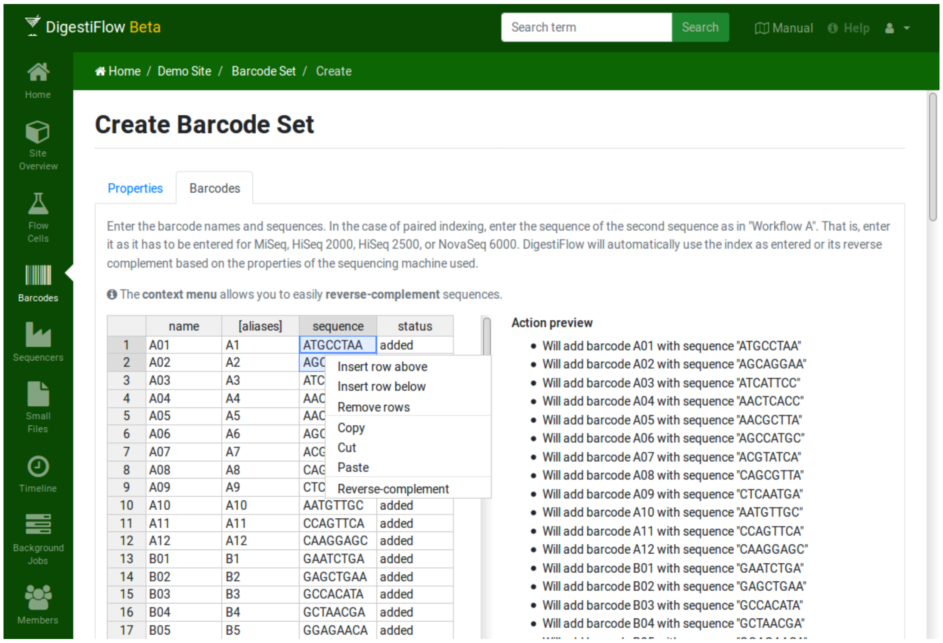

	name	[aliases]	sequence	status
1	A01	A1	ATGCCTAA	added
2	A02	A2	AGC	
3	A03	A3	ATC	
4	A04	A4	AAC	
5	A05	A5	AAC	
6	A06	A6	AGC	
7	A07	A7	ACG	
8	A08	A8	CAG	
9	A09	A9	CTC	
10	A10	A10	AATGTTGC	added
11	A11	A11	CCAGTTCA	added
12	A12	A12	CAAGGAGC	added
13	B01	B1	GAATCTGA	added
14	B02	B2	GAGCTGAA	added
15	B03	B3	GCCACATA	added
16	B04	B4	GCTAACGA	added
17	B05	B5	GGAGAACA	added

Fig. 3: The editor tab for editing the barcodes.

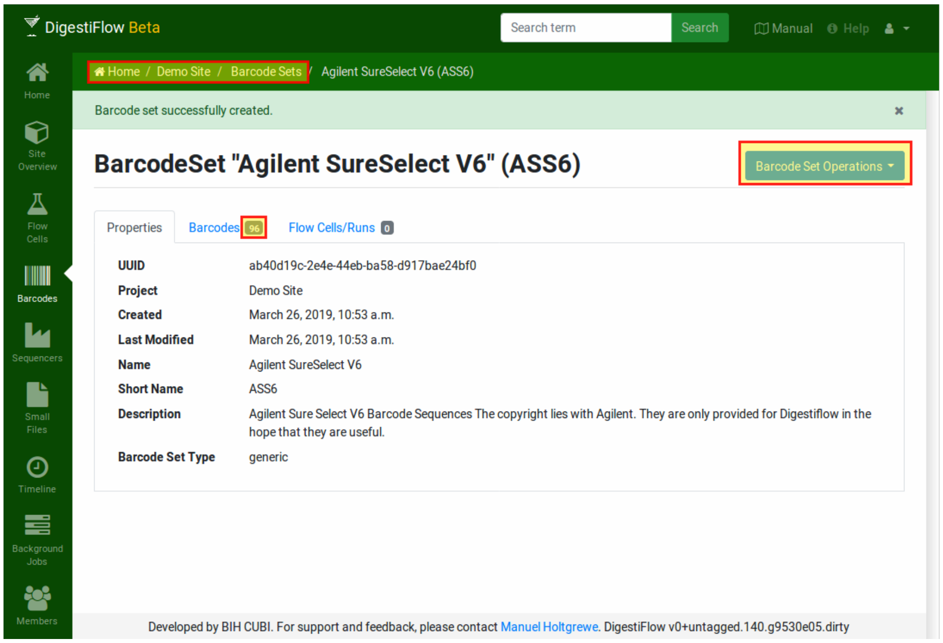

Fig. 4: The barcode set detail view shows the basic properties and has a tab for displaying the barcode set's barcodes.

CHAPTER 6

Tutorial: Flow Cells

Click on the "Flow Cells" icon in the left action menu. Here, you get a list of the flow cells in your site. We will first create a new flow cell manually and then use some auto-generated data for showing you the automated import with Digestiflow CLI.

6.1 Manual Flow Cell Creation

Click the blue "Flow Cell Operations" button on the top left to create a new flow cell. You see the flow cell editor form with tabs "Properties" and "Sample Sheets".

The "Properties" tab allows you to set the basic properties.

Name The name of the flow cell folder as you would enter it when starting your sequencing process, e.g., `160303_ST-K12345_0815_A_BCDEFGHIXX_LABEL`. This would describe a flow cell with Illumina ID `BCDEFGHIXX` run on March 3, 2016, on the machine with the vendor ID `ST-K12345` in its 815th run in slot `A` having a manual label of `LABEL`. Note that for NextSeq, the slot `A` is often unseparated with the flow cell ID.

Manual Label An optional manual override of the label to use in Digestiflow Server.

Description An optional short description of the flow cell, e.g., that it is a repetition of earlier drop-outs.

Num Lanes Number of lanes on flow cell.

Sequencer Operator Name of the sequencing operator.

Demultiplexing Operator Name of person responsible for demultiplexing.

Status Sequencing Current state of sequencing step.

Status Conversion Current state of demultiplexing or archive creation step.

Status Delivery Current state of data delivery.

Delivery Type The delivery types, either sequencers, archive of base calls, or both.

Barcode Mismatches Optional, specification of the number of mismatches.

Planned Reads Planned read specification when programming the sequencer, Picard-style. A sequence of number-letter pairs, e.g., `150T8B8B150T` (for 150T, 8B, 8B, 150T representing 150 template bases, 8 barcode bases, 8 further barcode bases, 150 further template bases). The allowed letters are `T` for template, `B` for barcode, `M` for molecular barcode, and `S` for skipping bases.

Current Reads Currently sequenced cycle sequence.

Demux Reads An optional override to use for demultiplexing. For example, if planned reads is `100T`, specifying `10M90T` specifies that the first ten bases are a molecular barcode ligated to 90 bases of template. As another example, if planned reads is `100T10B10B` then `100T10B10M` specifies 100 template reads, tenbarcode reads (used for demultiplexing), followed by ten further molecular barcode bases (that will be handled just like another read).

The tab "Sample Sheet" allows for editing the sample sheet. The columns are:

name The name of the library.

project ID An optional project identifier.

organism The organism of the sample.

i7 kit The kit used for the i7 barcode.

i7 sequence The actual sequence. An entry from the selected i5 kit, or a DNA sequence if `type barcode -->` was selected. Always enter the forward sequence, the demultiplexing process will reverse-complement depending on the dual indexing workflow. When doing copy-and-paste from your Excel sheets, you can either use the official adapter name or one of its aliases.

i5 kit The kit used for the i5 barcodes.

i5 sequence The actual i5 sequence, see notes for i7 sequence.

lane(s) The lanes that the library was sequenced on. Can be given as a single number, e.g., `1`, as a comma-separated list, e.g., `1,2,4`, a range `1-4`, or as a mixture `1,4-7`.

custom cyles Optional specification of per-library "Demux reads" cycle settings.

Once you have edited the sample sheet to your liking, click "Create" on the bottom right.

6.2 Automated Flow Cell Creation

Alternatively to entering the flow cell manually, you can also use Digestiflow CLI to automatically import the flow cell.

6.2.1 Installing Digestiflow CLI

Digestiflow CLI is best installed using conda/bioconda. The installation of bioconda is described in the bioconda manual. For example, for Linux:

```
# wget https://repo.anaconda.com/miniconda/Miniconda3-latest-Linux-x86.sh
# bash Miniconda3-latest-Linux-x86.sh -b -p $PWD/miniconda3
# source miniconda3/bin/activate
# conda config --add channels defaults
# conda config --add channels bioconda
# conda config --add channels conda-forge
# conda install digestiflow-cli
```

6.2.2 API Tokens

Next, you will need to get an API token. This token allows you to use clients of Digestiflow clients such as CLI and Demux without saving your password in clear text.

To obtain a token click on the little person icon on the top left of Digestiflow. Then, click "API Tokens", "Token Operations", "Create", "Create", and copy the token. You will not be able to see it again.

Next, create a `.digestiflowrc.toml` file in your home directory for configuration.

```
cat >~/.digestiflowrc.toml <<EOF
# Use 4 threads by by default.
threads = 4

[web]
# URL to your Digestiflow instance. "$url/api" must be the API entry URL.
url = "https://flowcells.example.org"
# The secret token to use for the the REST API, as created through the Web UI.
token = "secretsecretsecretsecretsecretsecretsecretsecretsecretsecretsecr"

[ingest]
# Create adapter histograms by default.
analyze_adapters = true
EOF
```

Then, update the following configuration settings:

`web:url` Adjust the API URL to point to the location where you are now running your Digestiflow Server instance.

`web:token:` Enter the token you received through the API into the file. Don't worry if you lose a token, you can always create a new one and delete old ones.

6.2.3 Using Digestflow CLI

Finally, we are ready to use Digestiflow CLI. You can use a command line like the following to scan all directories below `$path`. A watcher task can then simply be setup by running this in a loop (e.g., using `sleep 5m` to avoid unnecessary I/O and API load) or using a cron job (in the case of cron, make sure to use appropriate lock files in your script). You can copy the site UUID by going to the project overview and copy-and-paste the string after `/project/` in your adress bar; it should look like `xxxxxxxx-xxxx-xxxx-xxxx-xxxxxxxxxxx`.

```
# digestiflow-cli ingest $path/*
```

The Digestiflow Server Git repository contains a helper script for creating fake flow cell data. You can call it as follows to create a directory with very few reads simulating HiSeq 2000 properties. Alternatively, you can extract the archive `130820_CSSIM_0123_B_TESTEST_Test_Label.tar.gz` with pregenerated data.

```
# cd tutorial
# bash fake-bcl-folder.sh -c 1000 hiseq 130820_CSSIM_0123_B_TESTEST_Test_Label
### OR
# tar xf 130820_CSSIM_0123_B_TESTEST_Test_Label.tar.gz
```

Now, we can actually use digestiflow-cli to import this flow cell. Make sure to use the correct project UUID when importing a flow cell directory.

```
# digestiflow-cli ingest --project-uuid xxxxxxxx-xxxx-xxxx-xxxx-xxxxxxxxxxx 130820_
↪CSSIM_0123_B_TESTEST_Test_Label/
Mar 26 09:37:12.961 INFO Running: digestiflow-cli-client ingest
```

(continues on next page)

(continued from previous page)

```
Mar 26 09:37:12.961 INFO Options: Settings { debug: false, verbose: false, quiet:␣
↪false, threads: 0, seed: 42, log_token: false, web: Web { url: "http://127.0.0.
↪1:8000" }, ingest: IngestArgs { project_uuid: "d4b303c4-0bca-46fe-b157-9c57ff86a628
↪", path: ["130820_CSSIM_0042_B_Test_FAKE_DATA/"], register: true, update: true,␣
↪analyze_adapters: false, force_analyze_adapters: false, post_adapters: true,␣
↪operator: "", sample_tiles: 1, sample_reads_per_tile: 1000000, skip_if_status_
↪final: true, min_index_fraction: 0.001 } }
Mar 26 09:37:12.979 INFO Starting to process folder "130820_CSSIM_0123_B_TESTEST_Test_
↪Label/"...
Mar 26 09:37:12.979 INFO Guessed folder layout to be MiSeq
Mar 26 09:37:12.979 INFO Parsing XML files...
Mar 26 09:37:13.091 INFO Registering flow cell...
Mar 26 09:37:13.233 INFO Done registering flow cell.
Mar 26 09:37:13.233 INFO You asked me to not analyze adapters.
Mar 26 09:37:13.233 INFO Done processing folder "130820_CSSIM_0042_B_Test_FAKE_DATA/".
Mar 26 09:37:13.234 INFO All done. Have a nice day.
```

Now, you should see a new flow cell in your Digestiflow Server instance after clicking the "Flow Cells" icon on the left. Note how the little yellow icon on the left indicates an issue with the flow cell (the sample sheets are missing). The little hourglass below the little beaker indicates that the sequencing has been found to be still running by Digestiflow CLI. The little asterisks below the chip and truck indicate that demultiplexing and delivery have not been started yet. The little files indicate that demultiplexing is desired but packing into an archive is not. The other icons indicate that no comment has been nset, no messages been added, and no files have been posted. The adapter index sequences have been analyzed, however.

Next, create the marker file for the run being complete and call `digestiflow-cli ingest` again. This will mark the flow cell as complete with a little green hour glass.

```
# touch 130820_CSSIM_0123_B_TESTEST_Test_Label/RTAComplete.txt
# digestiflow-cli ingest --project-uuid xxxxxxxx-xxxx-xxxx-xxxx-xxxxxxxxxxx 130820_
↪CSSIM_0123_B_TESTEST_Test_Label/
[...]
[...] rta_version: 2, status_sequencing: "complete", status_conversion: "initial" [...
↪]
[...]
```

Clicking on the flow cell fake vendor ID "TESTEST" takes you to an overview of the flow cell's properties overview.

The icons next to "Sample Sheet" and "Index Stats" indicate missing or inconsistent data as displayed when moving your mouse cursor over them. Here, the sample sheet is yet empty while the index stats contain entries that cannot be found in the (empty) sample sheet.

In the next step, we will fill the flow cell's sample sheet.

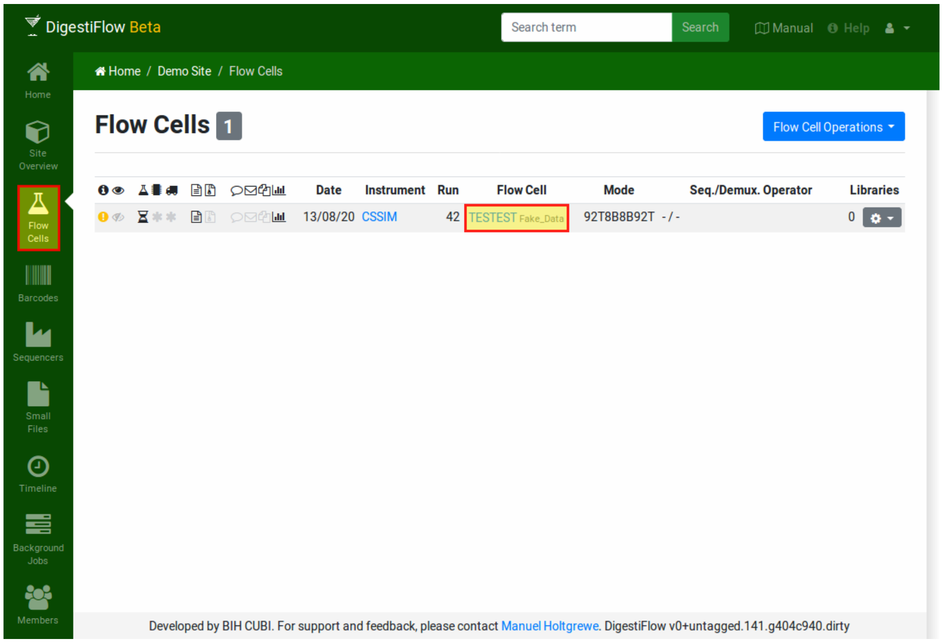
DigestiFlow Beta
Search term
Search
Manual
Help
Home / Demo Site / Flow Cells
Home
Site Overview
Flow Cells
Barcodes
Sequencers
Small Files
Timeline
Background Jobs
Members
Flow Cells 1
Flow Cell Operations
Date Instrument Run Flow Cell Mode Seq./Demux. Operator Libraries
13/08/20 CSSIM 42 TESTEST Fake_Data 92T8B8B92T - / - 0
Developed by BIH CUBI. For support and feedback, please contact Manuel Holtgrewe. DigestiFlow v0+untagged.141.g404c940.dirty

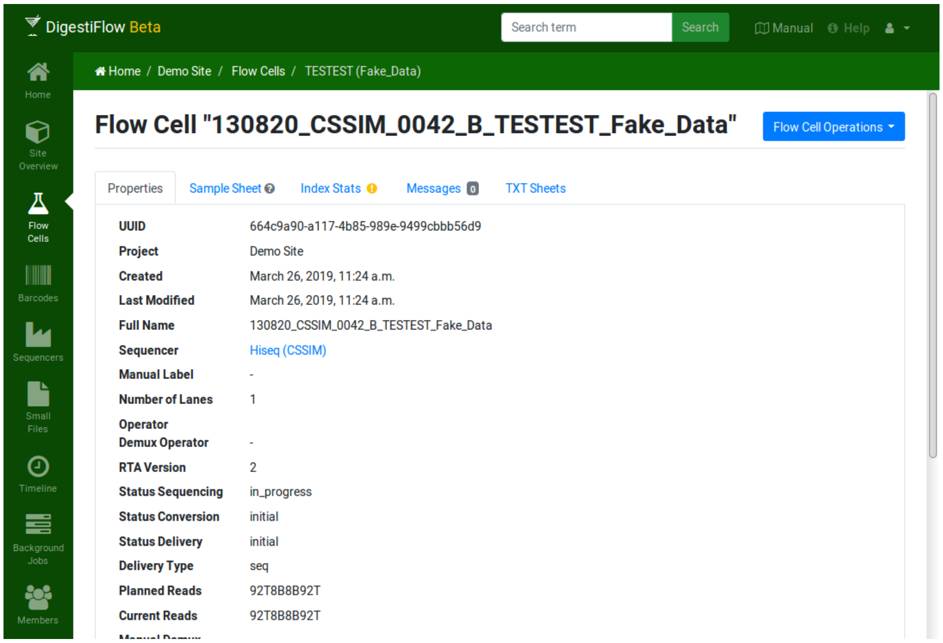
DigestiFlow Beta
Search term
Search
Manual
Help
Home / Demo Site / Flow Cells / TESTEST (Fake_Data)
Flow Cell "130820_CSSIM_0042_B_TESTEST_Fake_Data"
Flow Cell Operations
Properties
Sample Sheet
Index Stats
Messages 0
TXT Sheets
UUID 664c9a90-a117-4b85-989e-9499cbbb56d9
Project Demo Site
Created March 26, 2019, 11:24 a.m.
Last Modified March 26, 2019, 11:24 a.m.
Full Name 130820_CSSIM_0042_B_TESTEST_Fake_Data
Sequencer Hiseq (CSSIM)
Manual Label -
Number of Lanes 1
Operator
Demux Operator -
RTA Version 2
Status Sequencing in_progress
Status Conversion initial
Status Delivery initial
Delivery Type seq
Planned Reads 92T8B8B92T
Current Reads 92T8B8B92T

CHAPTER 7

Tutorial: Fill Out Flow Cell

It is very common that the wet-lab curates spreadsheets for the libraries, annotating the sample/library names with the used barcode and measurements results such as concentration or quality control results. It is thus desirable to re-use and copy-and-paste this information instead of re-entering it. We will thus assume this use case for this tutorial.

7.1 The Sample Spreadsheet

Download the file `130820_CSSIM_0123_B_TESTEST_Test_Label.xlsx` from here and open it. This file contains an excerpt of the information that the authors would expect to get from the wet lab. Open the file with your favourite spreadsheet program and have a quick look. The file is simple enough, containing the sample name, the i7 and i5 index names as well as the lane numbers on which the sample were loaded.

7.2 Transfer into Digestiflow

You can access the flow cell editor using the blue button on the flow cell detail view or by clicking the little gray icon next to the entry for the test flow cell and then clicking "Update". Select the "Libraries" tab to display the flow cell library editor. You will now copy over the data column-by-column from the sample sheet.

1. Select the cells with the library names in your spreadsheet program. Copy them into your clipboard (e.g., using Ctrl+C). Insert them into the libraries table in Digestiflow by selecting the first cell in the "sample names" column and then either pressing Ctrl+V or right-clicking on the cell and clicking "paste".
2. All data is of human origin. Select the top-most cell of the "organism" cell and select "human" after clicking the little triangle on the right side of the cell. Then make sure the cell and select the cell content using Ctrl+C or right-click and selecting copy. Select the cells in the same column up to the row with the last sample name. Then paste the value to all cells using Ctrl+V or right-clicking and selecting "paste".
3. Next, select the correct i7 barcode index **types** using the little triangle as in step 2. Then use the same approach to copy and paste this values to all cells in this column.
4. Next, we can copy and paste over the i7 barcode **names** names into the next column using the same approachas in step 1.

5. Repeat the step for setting the i5 barcode index type and names as you did for the i7 barcodes in step 3 and 4.
6. Finally, copy over the lane numbers from the spreadsheet app as you did with the library names.

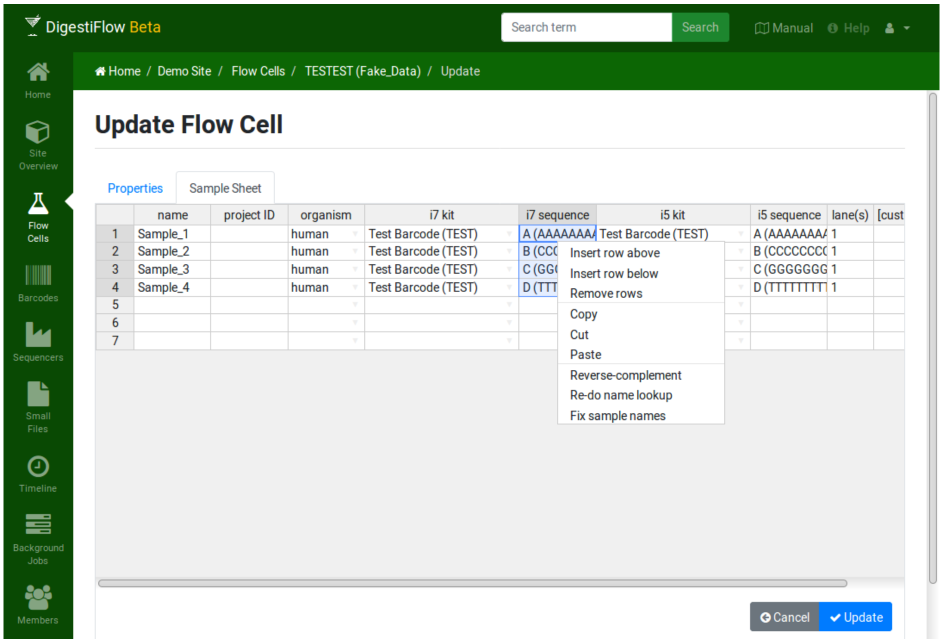

Fig. 1: The filled out sample sheet with the generated example data.

Note that there are various actions available (such as deleting a row of the table) in the context menu that opens when right-clicking on a cell. Don't forget to save your changes using the "Save" button once you are happy with the libraries table.

CHAPTER 8

Tutorial: Demultiplex Flow Cell

The final step is to perform the actual demultiplexing of the flow cell's base call data into FASTQ sequences. The first (easy) step is to install the `digestiflow-demux` program using Conda. The second (also easy) step is to perform the `digestiflow-demux` call that will handle everything else. The third step… there is no step three!

8.1 Installing Digestiflow Demux

After activating your Conda installation (if you have not done so already), you can simply install the `digestiflow-demux` conda package into its own environment.

```
~ # source path/to/miniconda3/bin
(base) ~ # conda create -n digestiflow-demux digestiflow-demux
[...]
(base) ~ # conda activate digestiflow-demux
(digestiflow-demux) ~ # digestiflow-demux --help
[...]
```

8.2 Performing the Demultiplexing

After having properly configured the `.digestiflowrc.toml` file earlier in the tutorial, Digestiflow Demux is already properly configured. All you have to do is to pass the proper project ID and the path to the flow cell directories to look at. Digestiflow Demux will then perform the demultiplexing, quality control, and post the results to the flow cell's messages in Digestiflow Server. Make sure that you have activated your conda installation and the `digestiflow-demux` environment as described above.

There is one more step before calling `digestiflow-demux` though. You have to set the "demultiplexing" status to "ready" in Digestiflow Server. Navigate to the flow cell list. Then, click on the state icon in the "demultiplexing" column and select "ready". The row will change to display a light blue sand clock icon to indicate that the demultiplexing process is ready to start.

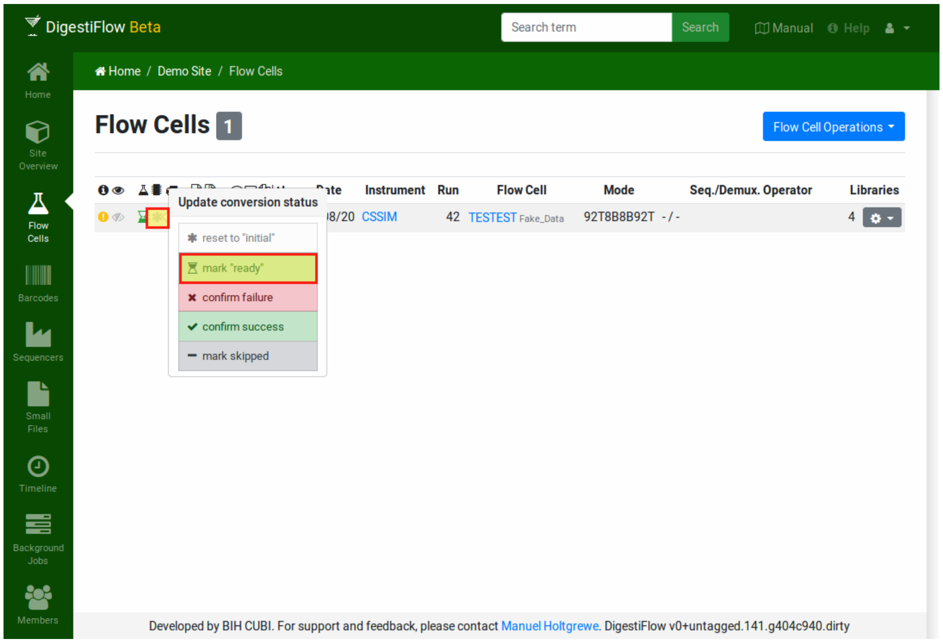

Fig. 1: Mark the flow cell as ready for demultiplexing by first clicking on the icon in the demultiplexing state column. Then select the "ready" entry in the popup.

Now you can start the demultiplexing using the `digestiflow-demux` program. Digestiflow Demux will only consider flow cells that are marked as "ready". If you want to restart demultiplexing after it fails or if you want to repeat it then you have to set the state to "ready" again as well.

Note: Although you would **usually not use it**, you have to use the `--with-failed-reads` argument for the synthetic example data set. The reason is that the adapter for the fourth sample is `TTTTTTTT` and this is filtered by `bcl2fastq` as a common artifact.

```
~ # source path/to/miniconda3/bin
(base) # conda activate digestiflow-demux
(digestiflow-demux) ~ # digestiflow-demux --help
[...]
(digestiflow-demux) ~ # mkdir -p demux-out
(digestiflow-demux) ~ # digestiflow-demux \
    --with-failed-reads \
    --project-uuid xxxxxxxx-xxxx-xxxx-xxxx-xxxxxxxxxxx \
    demux-output/130820_CSSIM_0123_B_TESTEST_Test_Label
    130820_CSSIM_0123_B_TESTEST_Test_Label
```

After demultiplexing was started, reloading the flow cell table will show a black sand clock for the demultiplexing of your flow cell. This indicates that the demultiplexing is running. Once complete a green sand clock will be displayed and on error a red one will be shown. You can confirm success or failure by clicking the flow cell and selecting the appropriate option. Note that in case of problems, the demultiplexing status will be updapted to "failed" by Digestiflow Demux. You will have to manually set it back to "ready" such that the flow cell is considered again for demultiplexing.

Note: The authors consider it best practice to use this feature after inspecting quality control results and/or logs to validate that the demultiplexing succeeded. In case of confirming failure and "giving up", the authors recommend to

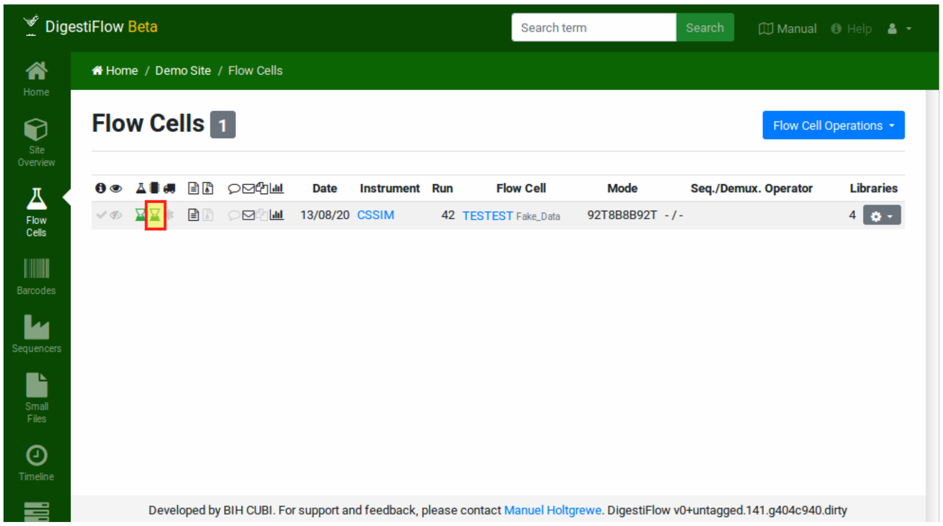

Fig. 2: The flow cell status after demultiplexing was complete.

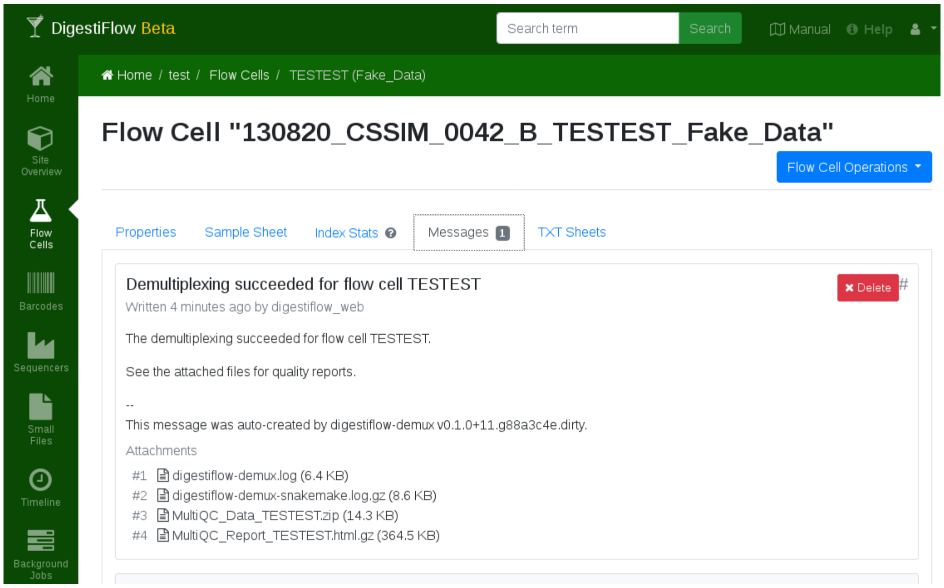

Fig. 3: The flow cell message with log and qc attachments after demultiplexing was complete.

leave a note in the flow cell's messages section explaining the failure and abandoning demultiplexing.

The demultiplexing and quality control should succeed. You should see according log messages and quality control results in the messages section of your flow cell.

CHAPTER 9

Permissions

Clicking the little "Members" icon in the left bar gives access to the Site member management area.

Note: When using LDAP authentication, users are only created in the Digestiflow database after the first login. That is, any user from your LDAP directory will be able to login and then has to be given access to a site as described here. This is a two step process and the user can only be added **after** logging in for the first time. Alternative, you can use the "invite user" feature described below to invite users that are not part of the project yet and select a role they should be given. After logging in for the first time, they will be granted site membership with the role that you selected. When using local users, you can add users through the Django administration interface.

9.1 Member Roles

Site members can have one of three roles.

Site Owner There can be one site owner that is able to modify all aspects of the site and the contained objects. Further, only the owner can assign the delegate role.

Delegates Users with this role also are able to modify all aspects of the site except for assigning delegate roles. They can add users with the contributor and guest role, though.

Contributor Contributor users are able to create and edit sequencing machines and barcode sets as well as flow cells.

Guests Site guests have read-only access to a site.

Further, standard Django user management allows to assign another important flags to a user (see below for details).

Superuser A user that is automatically granted all permissions within the full Digestiflow installation.

9.2 Member List

On this page you can see the list of users that have access to the site and their role within the project. User the blue "Member Operations" button to access the functionality to add or invite members, and view the invites. Next to each entry, the little gray button gives access to updating or deleting users. Of course, a confirmation dialogue will be display before removing a user from a project.

9.3 Managing Memberships

Managing site memberships should be self-explanatory.

9.4 Sending Invites

The main limitation that is faced when using LDAP authentication is the fact that users have to log in first in order to create a user record in the Digestiflow Server database. The onboarding process of users can be simplified by the invite feature. Here, you send an invitation email to a users with a link for logging into a given Digestiflow site. When sending the invite you also define a role for the user. Upon accepting the invitation by clicking on the link and following the instructions, the user will be automatically granted the appropriate role on the project (this does not work for project owners yet).

9.5 Django Admin

Super users can also access the Django administration interface. Django is the web application framework used for developing Digestiflow. You can access is through the User menu.

Note: Note that using the admin interface beyond managing users can damage the data in your Digestiflow installation.

After opening, scroll down to the "Users" section and click the little "Users" link within for managing users. The interface is self-explanatory. The relevant features are:

Create Local Users Even when using LDPAP authentication, you can create local users in the databse. This is useful for creating single-purpose users, e.g., for isolating the import/demux process for each machine or site and decoupling it from personal accounts.

Reset Password You can update the password of a user in the Digestiflow database. Note that this only applies to local users.

Disabling Users Uncheck the `Active` box in the "Permissions" section of a user to disable users (they will not be able to login any more).

Superuser Check the `Active` box in the "Permissions" section of a user to give all permissions to this user.

Don't forget to apply the changes made to users by clicking "Save".

Note: The Django group and permission system is not used for Digestiflow.

CHAPTER 10

Instance-Wide Search

You can search for any object in the Digestiflow database by using the little search box on the top of the page (given that your search term is longer than three characters).

The record fields considered in the search include the foll

- Barcode sequences of libraries on flow cells and in barcode sets.
- Flow cell, library, barcode set, barcode, and site names.
- Messages and the file names of any attachment.

Note that users are only able to find objects from the sites that they have access to.

CHAPTER 11

Sequencers

Clicking the little "sequencers" icon on the left-hand button bar takes you to the Sequencer Mangement Section.

11.1 Sequencer List

On the sequencer list page, you can see table with all sequencers. For each sequencer, the creation date, vendor ID, label, number of runs, and short description is show. Clicking on a sequencer's vendor ID leads to the detail page of the sequencer. The little gray button next to each entry gives access to updating and deleting sequencers. A confirmation dialogue will be displayed before actually deleting the record from the database. Note that deleting a sequencer is a permanent action that cannot be undone. Use the blue "Sequencer Operations" button on the top right to access functionality for creating new sequencers.

11.2 Sequencer Details

On the sequencer details page, you see two tabs. "Properties" displays the sequencer properties and "Flow Cells/Runs" that shows a list of all runs of this particular sequencer. Use the blue "Sequencer Operations" to access functionality for deleting and updating the sequencer.

The "Properties" tab shows the following information for each flow cell:

UUID The identifier used by Digestiflow for identifying the sequencer. This value is only used by the Digestiflow system.

Site The site that the sequencer is in.

Created The creation time of the sequencer record in the database.

Last Modified The last modification time of the sequencer record in the database.

Vendor ID The identifier assigned by Illumina to the sequencing machine. This value is part of the run folders (e.g., `NS501234` for a NextSeq 500 sequencer). **This is the most important configuration value.**

Label Short user-defined label for easily identifying the sequencer. You can specify any string here.

Description An optional, potentially longer description for the sequencer.

Machine Model The model of the sequencing machine. This value is only used for display of the sequencer in Digestiflow Server.

Slot Count The number of slots for flow cells. This is equal to the number of flow cells in your sequencer, e.g., `1` for NextSeq 500 and `2` for HiSeq 4000. This value is used for sanity checks when entering data manually into Digestiflow Server.

Dual Indexing Workflow The "Illumina dual indexing workflow" to use. **This is the second most important parameter.** The value will be used for automatically reverse-complementing the i5 barcode adapter when read as the second index read.

In the case of **Workflow A**, the i5 barcode adapter sequence is read in forward orientation. On the case of **Workflow B**, the i5 barcode adapter sequencer is read in reverse orientation. In the two sentences above, *forward* and *reverse orientation* are meant with respect to the sequence given in the sequencing chemistry manuals.

Workflow A is used by NovaSeq 6000, MiSeq, HiSeq 2500, and HiSeq 2000 while workflow B is used by iSeq 100, MiniSeq, NextSeq, HiSeq X, HiSeq 4000, and HiSeq 3000.

More details can be found on the Illumina Support website.

CHAPTER 12

Barcodes

Clicking the little “barcodes” icon on the left-hand button bar takes you to the Barcode (Set) Management Section.

12.1 Barcode Set List

On the barcode set list page, you can see the table with all barcode sets registered for the given site. For each bacode set, the creation date, name, short name, and desdription is shown. Clicking on the barcode set’s name leads to the detail page of the barcode set. The little gray button next to each entry gives access to updating and deleting barcode sets. A confirmation dialogue will be displayed before actually deleting the record from the database. Note that deleting a barcode set is a permanent action that cannot be undon. Use the blue “Barcode Set Operations” button on the top right to access functionality for creating new barcode sets.

12.2 Barcode Set Details

On the barcode set details page, you see two tabs “Properties” and “Barcodes”. “Properties” displays the barcode set properties and “Barcodes” that shows the list of all barcodes in this barcode set.

The “Properties” tab shows the following information for each barcode set:

UUID The internal identifier used by Digestiflow for identifying the barcode set. This value is only used internally by the Digestiflow system.

Created The creation time of the barcode set in the database.

Last Modified The last modification time of the barcode set record in the database.

Name The name of the barcode set, e.g., “Agilent SureSelect Human All Exon V6”.

Short Name A short name of the barcode set that is displayed together with the barcode name in flow cell visualizations.

Description A potentially longer description of the barcode set.

Type Either the type "generic" for normal barcode sequence or "10x Genomics Barcode" for 10x Genomics-style comma-separated barcode lists.

The "Barcodes" tab displays a table showing the following for each barcode.

Name The primary barcode name, e.g., `D501`.

Aliases An optional, comma-separated list of name aliases. E.g., you could use `501`, `1`, or `501,1` if the name is `D501` and you are getting wet-lab Excel sample sheets using `501` and `1` for the identification of the barcode.

Sequence The barcode sequence (in forward orientation as in the kit documentation for Illumina dual index workflow A). In the case of 10X Genomics style barcodes, this can be a list of comma-separated barcodes.

12.3 Updating Barcode Sets

The page for updating a barcode has two Tabs: "Properties" and "Barcodes". In the "Properties" tab you can update the basic properties of the barcode set such as its name. The "Barcodes" tab shows a spreadsheet-like view for updating the barcodes. In case of any display errors of the barcodes spreadsheet table simply click on the top-left cell which should make the spreadsheet table redraw properly. The table has a column for each of the barcode properties listed in *Barcode Set Details*. The last column indicates whether the row was changed, added, or is to be removed when saving.

Note that the table component behaves similar to spreadsheet software such as Excel in that you select ranges, drag down the current selection on the lower-right corner to copy its values or first select a range and then pasting (Ctrl+V) a copy of the current clipboard value to all selected cells. You can also copy a range of cells in another spreadsheet program such as Excel, select a cell in the Digestiflow barcode spreadsheet and then insert the cells there. In the case that the table has too few rows, new rows will be added automatically. Empty lines at the bottom are ignored.

Also note that various actions such as copying, pasting, inserting rows, and reverse-complementing the selected barcode sequences is available in the context menu. Simply right-click on any cell to show the context menu.

Do not forget to click "Save" for applying your changes.

CHAPTER 13

Flow Cell Overview

Flow cells are easily the most complex object in Digestiflow as they relate to the sequencer object that the flow cell was sequenced with, contain libraries and each library can relate to one or two barcodes used for them. Further, the sequencing, demultiplexing, and delivery state (cf. *Tutorial: Overview*) is modelled as flow cell attributes.

In the data model of Digestiflow, a "flow cell" actually means a "flow cell run", that is the information that the flow cell with a given ID was processed as the i-th flowcell in a given sequencing machine. It *is* possible to have a flow cell appear in two runs (and thus have two runs with the same flow cel ID), e.g., if the operator detects an error after starting the sequencer and immediately cancels the sequencing. Given the correct timing, the flow cell will remain unused and can be started again (with an incremented run number, though). As this case will be very rare (and the cancelled run does not generate any sequencing data), we will use the simplification of referring to "flow cell run" as "flow cell".

13.1 Run Directories

The sequencing devices store flow cell **run directories** with the following naming scheme. The different parts of the directories are separated by undescores.

```
                       run number on     flow cell
date/month/year          sequencer       vendor ID
    .----.                 .----.      .----------.
    |    |                 |    |      |          |
    YYMMDD_MACHINE-VENDOR_RUN-NO_SLOT_FLOW-CELL-ID_LABEL
           |             |        |  |              |   |
           '-------------'        '--'              '---'
         machine vendor ID    the slot can     user-defined label
                                be A or B         for flow cell
```

There are some caveats:

- The trailing underscore is missing for NextSeq (where the slot is always A), such that there is a field less and the flow cell ID field always starts with an A.
- The label might be missing (also no underscore), be empty (trailing undescore) and might contain whitespace.

13.2 RTA Files

The Illumina machines run a software called "RTA" (Run-Time-Analysis) that is responsible for operating the machine and everything from base calling, writing out statistics, storing thumbnail images, and displaying online quality control measures. The RTA also creates files describing the machine configuration and run parameters as well as the current sequencing state.

Digestiflow extracts its required metadata information from the sequencing output directory and the files created by RTA. This implies that only properly formatted run directories can be imported (if they deviate from the description above, Digestiflow CLI and Demux will not work properly).

13.3 Flow Cell Records

Each flow cell has various properties described in *Flow Cell Views* and refers to the Sequencer database object it was run on. This implies that you have to register all sequencers that you would like to in a given site. Note that the sequencer vendor ID has to be unique within each site and you have to register each sequencer individually in the site that you want to use it in.

Each flow cell contains a list of libraries. Each of these libraries has a name that is unique in the context of the flow cell. Further flow cells can have a primary and/or secondary barcode (or no barcode at all). These are the barcode that can be used for demultiplexing of the library. This can either refer to an barcode from an existing barcode set but it is also possible to specify a custom sequence for ad-hoc definition of barcodes. Each library is also specified to be loaded on one or more lines. Of course, each barcode (or barcode combination in the case of dual sequencing) can only occur once on each lane as demultiplexing would not be possible otherwise. Obviously, different libraries can use the same barcode sequence if they are loaded on different lanes.

13.4 Cycle Information

When starting the machine, the operator provides a certain program with cycle information to the machine. This specifies how many reads are created and whether a read is a template, an index barcode, or a molecular barcode. Some examples are given below (the commas can be omitted).

- `150T,10B,10B,150T` – 150 template bases, 10 barcode bases, 10 barcode bases, 150 template bases,
- `75T,10B,75T` – 75 template base, 10 barcode bases, 75 template bases,

This information can be overriden for the whole flow cell. E.g., if you have a flow cell that contains single-cell data, the second case might call for the following:

- `8M,2S,65T,10B,75T` – 8 molecular barcode bases, skip next 2 read bases, 65 template bases, 10 barcode basese, 75 template bases.

You can also override this information oin a per-library fashion. That is, you can mix libraries from different single-cell platforms. It is also possible to mix data from exome sequencing and low-input protocols such as Agilent SureSelectXT where the molecular barcode might be ligated in the same read position as the index barcode read in the exome library. This enables the efficient use of the ever-increasing sequencing capacity by using a high degree of multiplexing.

CHAPTER 14

Flow Cell Views

Differently from the rest of the manual, we will first describe the flow cell details view as this simplifies the explanation.

14.1 Flow Cell Details

The detail view shows the following information for each flow cell. This view also highlights errors that were detected in the sample sheet or inconsistencies between the sample sheet and the raw BCL calls.

Note that the messages tab is documented in detail in *Flow Cell Messages*.

14.1.1 Properties Tab

This tab shows the detailed information about the flow cells.

UUID The internal identifier used for the flow cell.

Site The name of the containing site.

Created Time of creation.

Last Modified Time of last modification.

Full Name Full folder name.

Sequencer The sequencer that this flow cell was run on. Links to the details view of the sequencer.

Manual Label Manual label override, if any.

Number of Lanes Number of lanes on the flow cell

Operator Name of the sequencing operator that was entered.

RTA Version The RTA version used for producing the flow cell.

Status Sequencing The status of the sequencing process.

Status Conversion The status of the base calling/base call archival process.

Status Deliver The status of the delivery process.

Delivery Type Delivery of sequences or raw base call archive.

Planned Reads Cycle configuration programmed into the sequencer.

Current Reads Currently processed reads.

Manual Demux Reads A manual override for the cycle information to use for demultiplexing.

Sheet Completeness Whether the sheet is completely filled.

All BCL indexes in sheet? Information about missing indices in sheet that were found in the BCL files.

All sheet libraries in BCL? Information about missing indices in bcl that were present in the sheet.

14.1.2 Sample Sheet

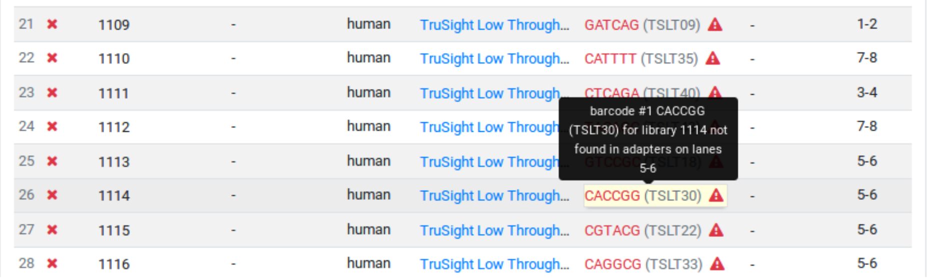

Fig. 1: Example of an error detected in the sample sheets. Some indices appear to be missing in the BCL data.

A list of the sample sheet with status indicators. Problems are indicated as yellow and red icons in the sample sheet. You can click on these icons to acknowledge the problem and silence it.

14.1.3 Base Call Information

This panel shows the index barcode statistics. For each lane and index read, this panel shows the distribution of barcode histograms. Erorrs and artifacts are highlighted. This view can be used for root cause error analysis of problems with demultiplexing.

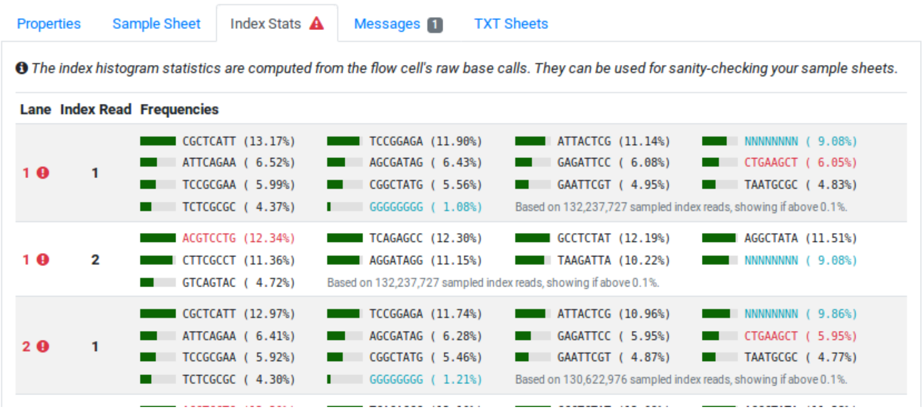

Fig. 2: Example of error display in the "Index Stats" tab.

In the example, libraries with barcodes CTGAAGCT, ACGTCCTG, and CTGAAGCT were found in the BCL data but were not present in the sample sheet. The sequence NNNNNNNN and GGGGGGGG were also found in the BCL data and not in the sample sheet. However, these are known artifacts and thus not marked as errors.

14.2 Flow Cell List

The flow cell list shows a list of all flow cells registered with the system. The information in each row is relatively dense to allow to get the most relevant information with a single glance and at the same time allow for easily updating the state for flow cells with very few clicks.

The columns are as follows:

Information / Notification In the case of any warning or error a yellow or red icon will be displayed here.

Observation Indicator This column indicates whether you are watching the flow cell and will receive emails on changes.

Sequencing Status, Demultiplexing Status, Delivery Status The first three columns display little icons for the three different states.

Deliver Sequence Conversion and/or Archives with Base Calls The next two icons indicate whether sequence conversion is asked and/or archives with the raw base calls are to be generated.

Comment / Message / Attachments Indicator Whether or not the comment field of the flow cell is filled, messages have been added to the flow cell and/or there are attachments with the messages.

Barcode Statistics Indicator Whether or not barcode statistics are available for the flow cell.

Date Sequencing date from the flow cell.

Instrument / Sequencing Machine The name of the sequencing machine. Clicking on the name leads to the detail view of the given sequencer.

Run Number The sequential number of runs on the given machine.

Flow Cell Vendor ID The vendor ID of the flow cell, label and potentially the manual label override

Mode The cycle configuration for the flow cell.

Seq./Demux. Operator Names of the demultiplexing operator.

Libraries Number of libraries in the sample sheet for the flow cell.

The little gray button on the right-hand side of the table rows allows to access the functions for updating and deleting flow cells.

14.3 Updating Status

When clicking the status icons, a window with a list of button appears. You can use this for changing the individual state of the flow cell with two clicks. After selecting the target state, the state will be updated and the row will be updated to reflect the results.

CHAPTER 15

Flow Cell Editor

The update view for flow cells has two tabs. The "Properties" tab allows to modify the basic attributes of the flow cell. The properties sequencing date, sequencing machine, slot, flow cell vendor ID and user-defined label are edited by setting the folder name. Note that you can define a **label override** different from the folder name for display in the flow cell list.

The "Libraries" tab allows for editing the libraries on your flow cell. The description of its functionality has two steps. First, we will explain the different components and how you can interact with them. This will allow you to enter a sample sheet library by library in the form. In most cases, the data is available in wet lab spreadsheet sample sheets. Digestiflow Server supports you in performing this task effectively and efficiently. The second step describes a workflow that the authors have successfully implemented in their daily work.

15.1 Editing Libraries

The libraries editor has the following columns.

Library ID The library identifier.

Organism (optional) The organism the library was generated from.

i7 Index Barcode Set The set of i7 barcodes to use or `enter your sequence here -->` if you want to enter a manual barcode.

i7 Barcode The i7 barcode sequence if using manually entered sequence. Otherwise, the barcode name and sequence will be displayed. If the barcode is not known in the barcode set, your cells will have a red background.

i5 Index Barcode Set The set of i5 barcodes, cf. "i7 Index Barcode Set" above.

i5 Barcode The i5 barcode sequence or barcode, cf. "i5 Barcode" above.

Lanes The lanes that the library was loaded on. You can mark a library to be loaded on multiple by lanes by using comma-separed lists and/or ranges, e.g., `1,2,4`, `1-4`, or `1-3,5-8`.

Cycle Configuration (Optional) Custom per-lane cycle configuration

15.2 Library Copy & Paste

This section describes the efficient workflow for copy-and-paste of data from spreadsheets.

As a prerequisite should have all your barcode sets prepared. The barcodes should either have the same name used in the sample sheet you are copying from or you should assign appropriate aliases. We assume that the source spreadsheet has a reasonable structure, e.g., it contains consecutive lines of libraries with separate columsn for sequence names, the i7 and i5 barcode index names, and lane numbers.

1. Copy the column with the library names from the spreadsheet into the library editor. For this, select the cell to start inserting into and then either press Ctrl+V or right-lick on the cell and select "paste".
2. Select the appropriate organism from the first row by clicking on the small triangle in the right part of the cell. Copy this value. Select the cells below the first one down to the one in the same or as the lowermost library name. Insert the organism value to fill out all selected fields. Adjust this step in case you have different organisms.
3. Select the type of the first barcode (if any) and use the same "copy single value and paste into range" step as in step 2. You can selcect `type your value here -->` for entering an ad-hoc barcode sequence.
4. Copy and paste the barcode names from your external sample sheet into the table using the same approach as in step 1. Note that if you have properly set the name and/or aliases of the barcodes then your barcode names will be automatically replaced by the string `{barcode name} ({barcode sequence}).` In the case that the lookup fails, the affected cells will be marked in red. If this does not work (e.g., you selected the wrong barcode set first, pasted non-matching barcode names, and then fixed the barcode set) then you can fix it as follows. Select the affected cell(s), right-click, and click "Re-do name lookup". This will re-start the resolution of barcodes from the barcode names. Note that lookup only works based on name not sequence.
5. If you have dual indexing then use the same approach as in step 3 for selecting the barcode.
6. Similarly, use the approach from step 3 for selecting/entering the second barcode.
7. Copy and paste the lane numbers for the library.
8. Optionally assign custom cycle configuration for the library if different from the cycle configuration of the flow cell.

Don't forget to save your changes.

Note that the editor will tolerate certain changes although it warns you by highlighting the fields in red. You will get warnings in the flow cell detail view.

CHAPTER 16

Flow Cell Messages

The "Messages" tab contains the user interface for posting messages, optionally with attachments.

On the top, you see a list of all messages. Messages can either consist of unformatted text (line breaks are interpreted) or Markdown. Markdown allows the embedding of links and highlighting of text. Each message can have a list of attachments.

The message system can be used for various functions.

1. The Digestiflow Demux tool will add a message to the flow cell with its results. In case of success, log files are attached as well as archives of the MultiQC report. In case of failure, only log files are attached.
2. You can use this for persisting the wet-lab Excel sheets that you base your Digestiflow sample sheets on.
3. Machine and demultiplexing operators can use this for persisting information and discussion results. E.g., in case of issues it can be useful to leave small notes that explain the issue. This can be followed by documenting the resolution. As you can find text in the message subjects and bodies, a site's messages quickly become a useful resource for issue root cause analysis. Instead of discussions disappearing in individual user's mailboxes, they are visible for all members of a site.

You can use the form at the bottom for writing messages. You can upload up to three attachments in one go. If you want to upload more attachments, click "save as draft". Afterwards, you will be able to more attachments (and also remove existing ones an modify the message subject or body). Each user can have at most one active draft for each flow cell.

Do not forget to finally send the message (or publish the draft) by clicking "Send".

CHAPTER 17

Admin Alerts

Users with superuser status have access to the entry “Alerts” in the user menu on th etop right hand side of the screen. This feature can be used for displaying messages that are visible to everyone (also publically on the login page). Common uses are for announcing maintenance times etc.

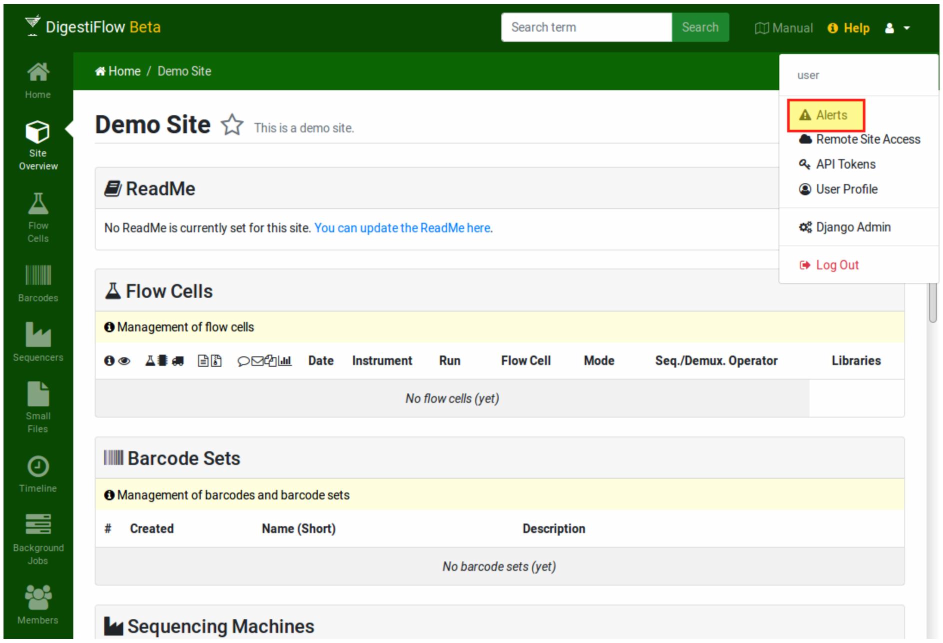

Fig. 1: The user menu displays the “Alert” entry for superusers. Use this link to access the the Admin Alerts administration section.

CHAPTER 18

API Tokens

In your "user menu", you will find a menu entry "API Tokens" that takes you to the site-wide API token management. An API token is a unique identifier for your account that consists of 64 randomly randomly characters and is thus infeasible to guess. You can use it for authenticating requests to the Digestiflow REST API in a secure manner that is independent of your password. Thus, you can store it in properly secured locations such as files that Digestiflow CLI and Demux can read.

Note: The major advantage of using tokens in APIs is that you don't have to store your password in plain-text files. Also, you can create an arbitrary number of tokens. Thus, in case a token is compromised, it can be exchanged easily (the same is true if you lose your token).

Note: For Digestiflow Cli and Demux, it also makes sense to create dedicated "machine" users in the Digestiflow Server database as described in the section *Django Admin*.

18.1 Listing API Tokens

Using the "API Tokens" in your "user menu", you will be directed to the "Tokens" list.

Here, you can see the list of currently active tokens. For each token, the table lists the creation time, expiry date, and a token "key" for identifying your token. On the right hand side of each token entry there is a little gray button that allows for the deletion of a token. Use the blue button "API Tokens" on the top right to access the token creation functions.

18.2 Creating API Tokens

When creating a token, the only thing that you can add is a "time to live." That is, tokens expire after a defined number of hours. Specifying `0` here makes the token live forever.

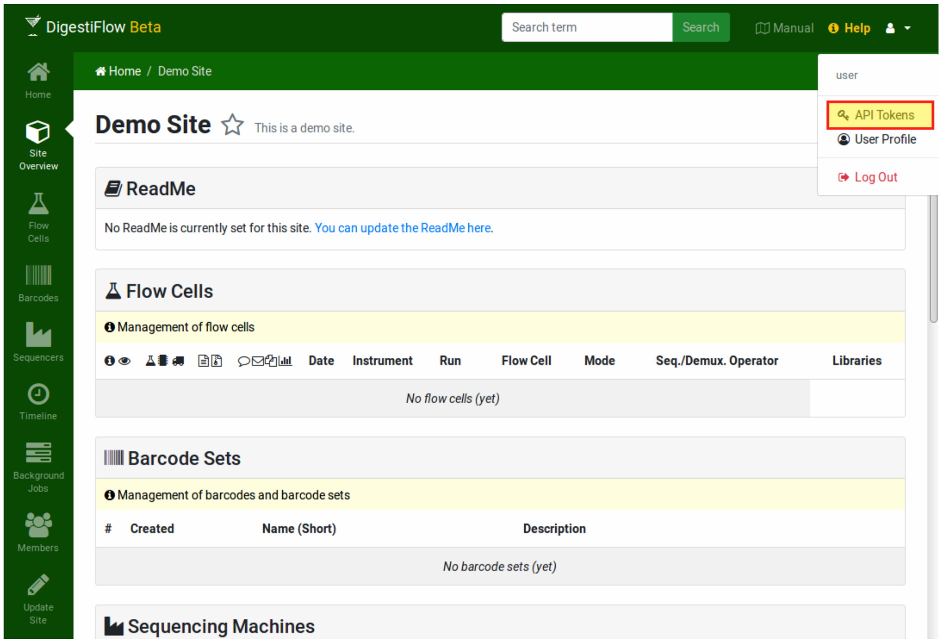

Fig. 1: The Link to the API token management in the user menu.

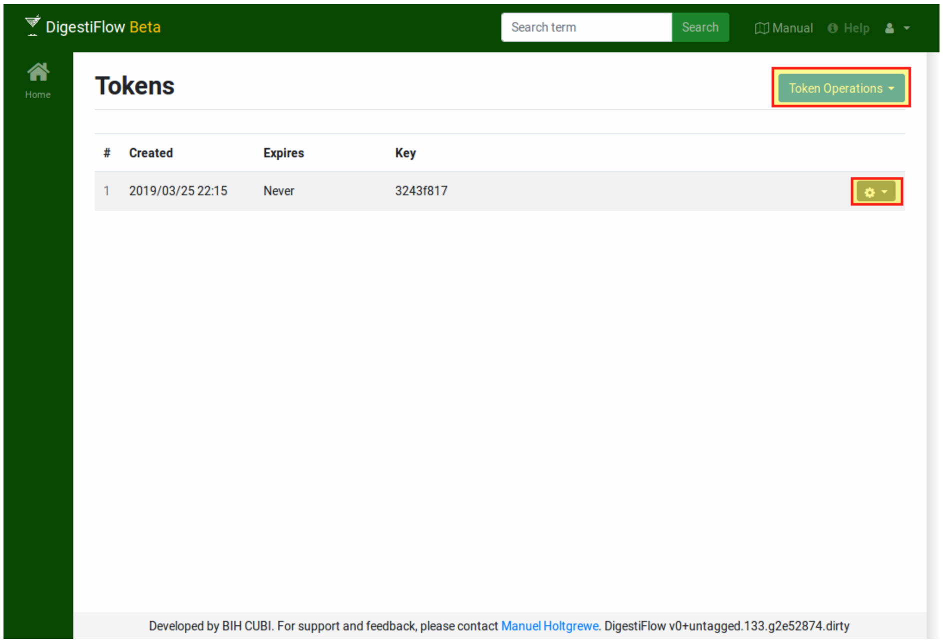

Fig. 2: The list of tokens for a user. Create tokens through “Token Operations” / “Create”, access the deletion function through the little gray icon on the left next to each entry.

After the token has been created you will be able to see it only once, so copy it into another file. In case you forget to copy the token don't worry as you can just create a new one.

CHAPTER 19

API Access

Digestiflow Server provides an API for managing all objects within a site: Sequencers, barcode sets, barcodes, flow cells, libraries, and messages with attachments. Generally, the API is available with the prefix `/api`.

The Digestiflow REST API is implemented using Django REST Framework which also allows for the easy exploration of the API in your browser. The same URLs that you can access in your browser can also be accessed by API clients or `curl` but will not render HTML pages but return JSON data.

Note: The Digestiflow REST API has not stabilized yet. The current version is considered to be "v0". After stabilizing as "v1.0", the API will use semantic versioning.

19.1 First Examples

The following assume you already have a first site, sequencer, barcode set, and flow cell setup already.

1. Quickly access the API by going to the detail view of your flow cell, click the blue "Flow Cell Operations" button and select "JSON export". The resulting output already! comes from the Digestiflow REST API.

2. Go to the flow cell list of your site. Copy the full URL and insert `/api` between the host name and `/flowcells` (*). From here, you can explore the API for managing flow cells.

3. Create an API token (see *API Tokens*) and copy it somewhere safe. You can now use the following `curl` command. `$URL` is the result of (*) in the example 2 and `$TOKEN` is the token.

   In the following example, the list of flow cells is empty in the given project.

   ```
   $ URL=http://127.0.0.1:8000/api/flowcells/${project_uuid}/
   $ curl -X GET $URL -H "Authorization: Token $TOKEN"
   []
   ```

19.2 Authentication

In the examples above you have already seen the two modes of authentication that Digestiflow REST API supports.

1. Use the browser cookie after logging into Digestiflow with a browser.
2. Use an API token with an appropriate header showing the API token to the server.

19.3 Object Identifiers

For understanding the API, you need to know that Digestiflow uses UUID keys (random strings, e.g., `3d4b6d4f-62a7-4534-9a08-b473adae7302`) for access. Each object has such an ID, including each site.

19.4 API Patterns

Generally, the basic API URLs have the following format (where `site` and `object` specify UUIDs):

1. `/api/<object type>/<site>/` (*) HTTP GET obtains a list of objects while POST allow to create new ones.
2. `/api/<object type>/<site>/<object>/` HTTP GET obtains the details of the object while POST allows for full updates, PUT allows for partial updates, DELETE allows deletion.

The easiest way to learn about the API is to navigate to the URL of type 1 (*) with your browser and explore it from here.

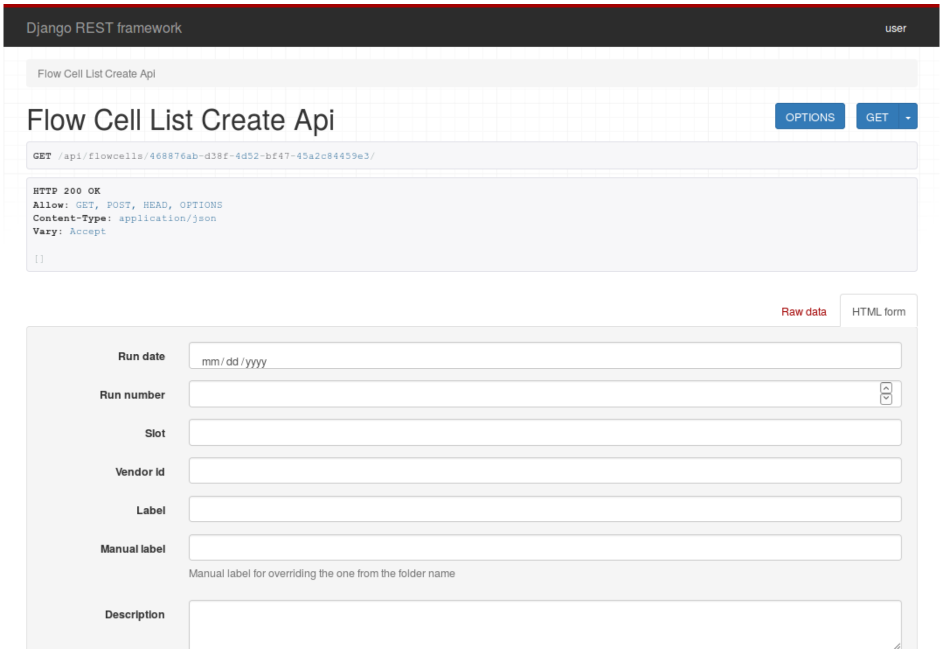

Fig. 1: The API detects if it is being accessed by a web browser. In this case, it renders a web page displaying the objects in pretty-printed JSON format. Also, it displays a form for manipulating the objects. In this case, an empty list of flow cells is being displayed to the user together with a form to create a new flow cell.

19.5 Available API Endpoints

/api/sequencers/<site>/ List sequencers or create new one in site.

/api/sequencers/<site>/<sequencer> Fetch, update, or delete sequencer.

/api/sequencers/by-vendor-id/<site>/<vendor ID> Lookup sequencer by its vendor ID.

/api/barcodesets/<site>/ List barcode set or create new one in site.

/api/barcodesets/<site>/<barcodeset> Fetch, update, or delete barcode set.

/api/barcodesetentries/<site>/<barcodeset>/ List barcode set or create new barcode in barcode set.

/api/barcodesetentries/<site>/<barcodeset>/<barcode> Fetch, update, or delete barcode.

/api/barcodesetentries/retrieve/<site>/<barcode> Fetch barcode using UUID without knowing its barcode set.

/api/flowcells/<site>/ List flow cell or create new one in site.

/api/flowcells/<site>/<flowcell> Fetch, update, or delete flow cell.

/api/flowcells/resolve/<site>/<sequencer vendor ID>/<run no>/<flowcell vendor ID> Fetch flow cell (run) from sequencer vendor ID, run number, and flow cell vendor ID.

/api/indexhistos/<site>/<flowcell>/ List index histogram records or create new one for flow cell.

/api/indexhistos/<site>/<flowcell>/<indexhistogram> Fetch, udpate, or delete single index histogram record.

/api/messages/<site>/<flowcell>/ List messages or create new one for flow cell.

/api/messages/<site>/<flowcell>/<message>/ Fetch, update, or delete message.

/api/attachments/<site>/<flowcell>/<message>/ List message attachments or create new one for message.

/api/messages/<site>/<flowcell>/<message> Fetch, update, or delete attachments.

CHAPTER 20

Contributors

(in alphabetic order)

- Dieter Beule
- Manuel Holtgrewe
- Clemens Messerschmidt
- Mikko Nieminen

CHAPTER 21

History / Changelog
